# Comparison of the effects of empagliflozin and glimepiride on endothelial function in patients with type 2 diabetes: A randomized controlled study

**DOI:** 10.1371/journal.pone.0262831

**Published:** 2022-02-16

**Authors:** Haruka Tamura, Yoshinobu Kondo, Kohei Ito, Masanori Hasebe, Shinobu Satoh, Yasuo Terauchi

**Affiliations:** 1 Yokohama City University School of Medicine Graduate School of Medicine, Endocrinology and Metabolism, Yokohama, Kanagawa, Japan; 2 Chigasaki Municipal Hospital, Endocrinology and Metabolism, Chigasaki, Kanagawa, Japan; Kurume University School of Medicine, JAPAN

## Abstract

Patients with type 2 diabetes who have cardiovascular disease and are receiving empagliflozin have a lower rate of primary composite cardiovascular outcomes. In contrast, glimepiride increases cardiovascular hospitalization when combined with metformin. Here, we assessed the effects of empagliflozin and glimepiride on endothelial function using flow-mediated dilation (FMD). In this prospective, open-label, randomized, parallel-group study, 63 patients with type 2 diabetes received metformin and insulin glargine U100 for 12 weeks. This was followed by additional treatment with empagliflozin or glimepiride for 12 weeks. The primary outcome was the change in the FMD measurement (ΔFMDs) at 24 weeks of additional treatment. Secondary outcomes comprised changes in metabolic markers and body composition. The empagliflozin group (*n* = 33) and glimepiride group (*n* = 30) showed no significant differences in ΔFMDs (empagliflozin, −0.11 [95%CI: -1.02, 0.80]%; glimepiride, −0.34 [95%CI: -1.28, 0.60]%; *P* = 0.73). Additionally, changes in glycated hemoglobin were similar between the two groups. However, a significant difference in body weight change was observed (empagliflozin, −0.58 [95%CI: -1.60, 0.43] kg; glimepiride, 1.20 [95%CI: 0.15, 2.26] kg; *P* = 0.02). Moreover, a body composition analysis revealed that body fluid volume significantly decreased after empagliflozin treatment (baseline, 35.8 ± 6.8 L; after 12 weeks, −0.33 ± 0.72 L; *P* = 0.03). Hence, although empagliflozin did not improve endothelial function compared with glimepiride for patients with type 2 diabetes, it did decrease body fluid volumes. Thus, the coronary-protective effect of empagliflozin is not derived from endothelial function protection, but rather from heart failure risk reduction.

**Trial registration:** This trial was registered on September 13, 2016; UMIN000024001.

## Introduction

Diabetes patients present a particularly high cardiovascular risk, making early detection of vascular failure essential. Hence, assessment of coronary endothelial vasoreactivity has important diagnostic and prognostic implications for patients with diabetes. An estimate of arteriosclerosis and the risk of cardiovascular events can be obtained by measuring endothelial dysfunction [1]. The flow-mediated dilation (FMD) method evaluates endothelial function in a noninvasive manner, based on the intrinsic ability of blood vessels to respond to blood flow [2]. FMD uses high frequency ultrasonographic imaging of the brachial artery to report on nitric oxide-induced vasodilation within the artery. Since FMD can predict cardiovascular events, it has been utilized in numerous investigations of arteriosclerosis [3–5]. However, variables such as age, systolic blood pressure, body mass index (BMI), sex, presence of diabetes mellitus, lipid-lowering medications, smoking, and a decrease in visceral adipose tissue mass can all impact FMD measurements [6–8].

Blood glucose levels in diabetes can be decreased by preventing proximal tubular glucose reabsorption and increasing urinary glucose excretion using an inhibitor of sodium glucose cotransporter 2 (SGLT2). Notably, such inhibitors can also reduce blood pressure [9] and body weight [9, 10]. The EMPA–REG OUTCOME trial revealed that treatment with empagliflozin, an SGLT2 inhibitor, reduced the risk of cardiovascular outcomes and death from all causes in patients with pre-existing atherosclerotic cardiovascular disease. However, the incidence of myocardial infarction or stroke did not differ significantly between the empagliflozin and placebo groups [11].

Furthermore, a meta-analysis showed that SGLT2 inhibitors improve the composite outcome of myocardial infarction, stroke, and cardiovascular death [12]. However, the effects varied according to the presence or absence of atherosclerotic cardiovascular disease. Indeed, these inhibitors could only reduce composite major adverse cardiovascular events in patients with existing atherosclerotic cardiovascular disease. Nonetheless, hospitalization following heart failure was reduced with SGLT2 inhibitor treatment regardless of atherosclerotic cardiovascular disease or heart failure status at baseline. However, the anti-atherosclerotic mechanism of SGLT2 inhibitors remains unclear [12].

In the UK Prospective Diabetes Study (UKPDS) 33 Group, microvascular complications were reduced with sulfonylurea therapy, however, no effect was observed on macrovascular disease. The risk of microvascular complications decreased following sulfonylurea treatment due to improved blood glucose control [13]. However, the observation that combined sulfonylurea and metformin therapy increased cardiovascular hospitalization or mortality was concerning [14]. The lack of an effect elicited by sulfonylurea on macrovascular disease may result from its inability to affect endothelial function. Considering that the reduction in microvascular complications associated with sulfonylurea treatment was derived from control of blood glucose and decreased glycated hemoglobin (HbA1c), we hypothesized that sulfonylureas might not improve endothelial function. Consequently, in this study we used FMD to compare endothelial function in patients with type 2 diabetes treated with either an SGLT2 inhibitor (empagliflozin) or a sulfonylurea (glimepiride).

## Materials and methods

### Study design

This study was registered with the University Hospital Medical Information Network Clinical Trial Registry (UMIN000024001) as a prospective, open-label, randomized, parallel-group comparison study. Approval for this study was obtained from the ethics committee of Chigasaki Municipal Hospital (approval No. 2017–08). The study protocol conforms to the provisions of the revised Declaration of Helsinki guidelines. Written, informed consent was obtained from all participating patients.

### Inclusion and exclusion criteria

Patients with type 2 diabetes (20–80 years of age) who were hospitalized for 1–2 weeks at the Chigasaki Municipal Hospital for diabetes education and blood glucose control were included in this study. Patients were placed on a controlled diet and treated with insulin following hospitalization. In patients already being treated for diabetes, prior treatments were discontinued and changed to insulin therapy upon patients being hospitalized for diabetes education and blood glucose control. Treatments were then changed to metformin and insulin glargine U100 at discharge. To attenuate glucotoxicity, patients received metformin and basal insulin therapy prior to discharge. All patients had a BMI ≤ 45 kg/m^2^.

Patients were excluded if they showed severe renal dysfunction (estimated glomerular filtration rate [eGFR] < 45 mL min^−1^ 1.73 m^−2^), liver dysfunction, were on steroid therapy, experienced cardiovascular disease and a cerebral infarction within 24 weeks of the study, had cancer, had severe infection, were traumatized, were or could become pregnant, were allergic to empagliflozin, insulin glargine U100, glimepiride, or metformin, or if the supervising doctor decided that the patient did not qualify for this study.

### Treatment and interventions

Patients were treated with metformin and insulin glargine U100 for only 12 weeks after discharge and were then randomized to receive additional treatment for another 12 weeks with either daily 10 mg empagliflozin or 0.5 mg glimepiride. HT enrolled participants and YK assigned participants to interventions. Patients were assigned enrollment numbers. The random allocation of patients was performed by YK at Chigasaki Municipal Hospital, and the assignment was blinded. According to Japanese guidelines, a 10 mg dose of empagliflozin is recommended as the initial starting dose. We selected a dose of 0.5 mg glimepiride, because this dosage exhibited an efficacy of lowering blood glucose that was similar to that of 10 mg empagliflozin [15–17]. Patients were randomly assigned in a one to one ratio. Randomization was stratified based on age, HbA1c, and FMD using the computed minimization method (MinimPy 0.3, Python Software Foundation). Blood samples were obtained in a fasting state. Each observation point involved two separate measurements.

Initial FMD measurements were made prior to randomization and additional treatment with empagliflozin or glimepiride. Subsequent measurements were made following additional treatment for 12 weeks. If fasting plasma glucose was maintained under 90 mg/dL after randomization, the insulin glargine U100 dose was decreased by one unit, once weekly. Otherwise, treatment was generally not changed following randomization. Patients were instructed to self-monitor blood glucose with a blood glucose system (ONE TOUCH Verio IQ, Johnson and Johnson Co., New Brunswick, NJ, USA) twice daily, as well as hypoglycemia symptoms. Hypoglycemia (i.e. blood glucose < 70 mg/dL) was ascertained using the values recorded by the patients.

### Flow-mediated dilation

FMD was measured using a UNEX EF38G (UNEXCorporation, Nagoya, Japan) by clinical technologists at the Chigasaki Municipal Hospital according to pre-published guidelines [2]. This automated edge detection system was used to measure the brachial artery diameter using high-resolution ultrasound. The pre- and post-artery diameters pressed by the cuff were measured. FMD was calculated using the following formula: FMD (%) = ([maximum diameter—diameter at rest]/diameter at rest) × 100.

### Endpoints and assessments

The primary outcome was the change in FMD, which was measured prior to, and following, 12 weeks of additional treatment (S1 Fig). Secondary outcomes included changes in the plasma levels of metabolic markers during the fasting state, which were measured prior to, and following, 12 weeks of treatment. Body composition components, such as skeletal muscle and total fat mass, as well as body fluid volume, were assessed using a multifrequency bioelectrical impedance analyzer (BIA; InBody720; InBody Co., Ltd., Seoul, South Korea) and they were included as the secondary outcomes. InBody 720 measures impedance of parts of arms, trunk, and legs with eight-polarities (two palm, thumb, anterior and posterior aspects of the sole) and six frequencies (1, 5, 60, 250, 500, and 1,000 kHz). Intracellular water (ICW), extracellular water (ECW), and total body water (TBW) were estimated. This accurately estimates total and appendicular body composition, independent of age and sex [18].

### Sample size and statistical analyses

The effect of SGLT2 inhibitors on endothelial function, as measured by FMD, was unknown at the time this protocol was developed. We estimated that the change in FMD measurement (ΔFMD) with empagliflozin treatment for 12 weeks would be 1.5 ± 2.0% based on the results from a previous study [19]. Considering a two-sided *P*-value of 5% and a power of 80%, we calculated that a sample size of 58 patients was required to detect a significant difference between the two treatment groups. We estimated that 10% of the enrolled patients were lost or did not meet the inclusion criteria. All data were analyzed based on the intention-to-treatment principle. The primary analysis was performed in the full analysis set (FAS), and robustness of the results was explored through sensitivity analysis in the per-protocol set (PPS). The repeated-measure endpoints were analyzed with linear mixed models that included intervention (empagliflozin or glimepiride), dummy variables for time (baseline or week 12), intervention-by-time interactions as covariates, and the subjects as a random effect. The covariance structure was a completely general (i.e., unstructured) covariance matrix. The results were reported as the least squares means with 95% confidence interval (CI) at each time-point. The analyses were performed using JMPPro12 (SAS Institute, Cary, NC, USA).

## Results

### Patients

**Fig 1:**
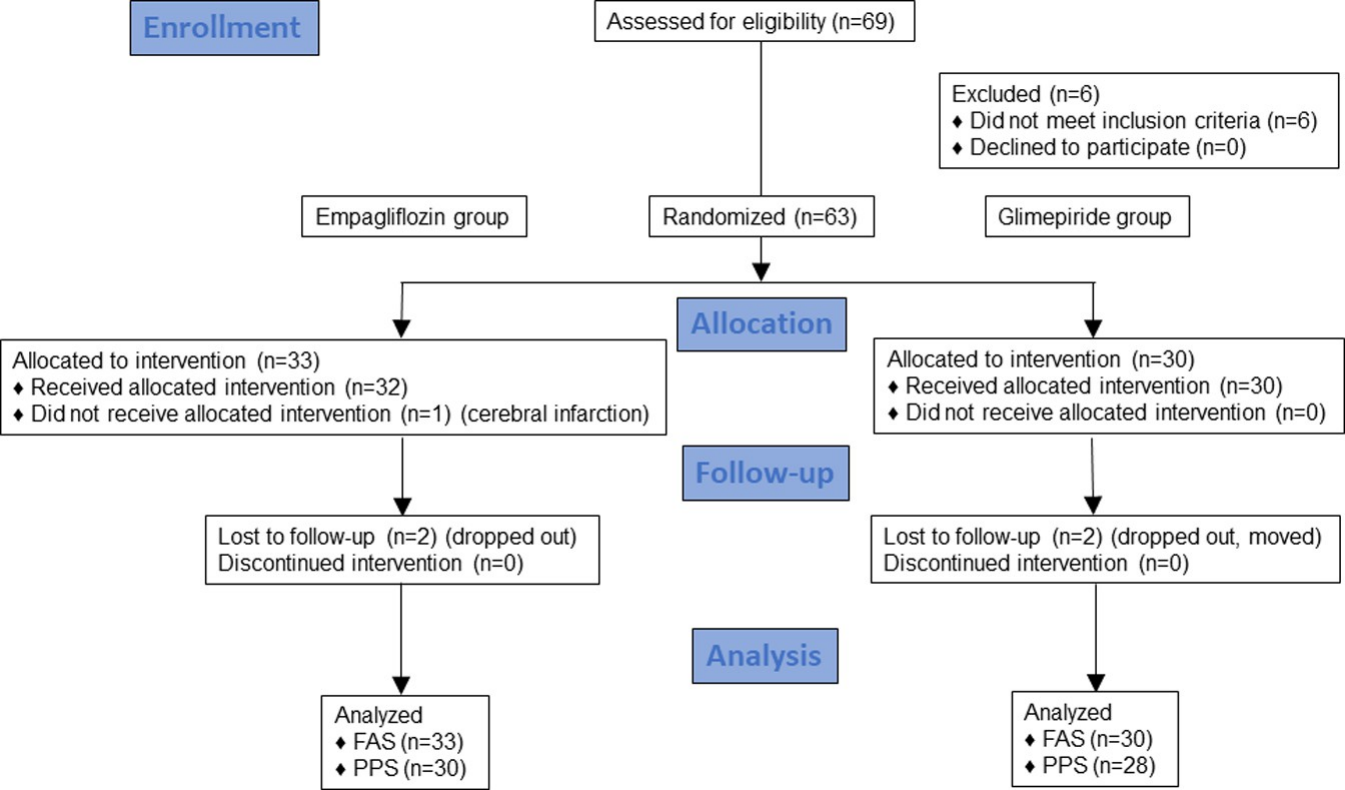
Consort 2010 flow diagram for patient selection. Ultimately, 33 patients in the empagliflozin group and 30 patients in the glimepiride group were analyzed.

Between June 2016 and 2018, 69 patients with type 2 diabetes were recruited for this study. The final date of recruitment was December 18, 2018, which was the date on which we achieved the target number of patients. The final follow-up date was June 14, 2019. A total of 63 patients were randomized at a one-to-one ratio to either the empagliflozin or glimepiride group. After study completion, 33 patients in the empagliflozin group and 30 patients in the glimepiride group were analyzed (Fig 1). The baseline clinical and biochemical characteristics in the two treatment groups were statistically similar (Table 1). Patients received the following drugs in the empagliflozin and glimepiride groups prior to enrollment in the study: metformin (21.2% vs. 13.3%, respectively); DPP-4 inhibitor (27.3% vs. 26.7%, respectively); SGLT2-inhibitor (6.1% vs. 3.3%, respectively); sulfonylurea (12.1% vs. 13.3%, respectively); α-glucosidase inhibitor (3.0% vs. 6.7%, respectively); glinide (0% vs 3.3%, respectively); insulin (15.2% vs. 6.7%, respectively); and a GLP-1 analog (0% vs 3.3%, respectively).

**Table 1:** Patient baseline characteristics.

	Empagliflozin group	Glimepiride group
	(± SD) *n* = 33	(± SD) *n* = 30
Age [years]	58.7 ± 8.8	53.8 ± 12.0
Sex (male/female)	21/12	20/10
Estimated duration of diabetes [years]	7.1 ± 8.1	4.6 ± 4.9
Recently diagnosed diabetes [%]	33.3	43.3
Current smoker [%]	24.2	40.0
FMD [%]	5.4 ± 2.0	5.4 ± 2.1
HbA1c [%]	7.0 ± 1.2	6.6 ± 0.7
FPG [mg/dL]	143.5 ± 66.6	125.6 ± 52.1
GA [%]	17.2 ± 3.7	16.3 ± 3.3
Body weight [kg]	69.9 ± 12.0	69.2 ± 16.6
BMI [kg/m^2^]	25.9 ± 3.9	25.7 ± 5.3
Cr [mg/dL]	0.76 ± 0.16	0.74 ± 0.17
eGFR [mL min^−1^ 1.73 m^−2^]	75.6 ± 12.7	81.5 ± 19.2
UA [mg/dL]	5.4 ± 1.1	5.4 ± 1.4
LDL-C [mg/dL]	95.7 ± 26.1	89.5 ± 27.8
HDL-C [mg/dL]	57.0 ± 17.7	58.1 ± 16.0
TG [mg/dL]	186.9 ± 102.4	171.4 ± 128.2
sBP [mmHg]	130.0 ± 15.9	130.9 ± 20.2
dBP [mmHg]	80.8 ± 10.2	78.6 ± 9.6
Metformin [mg]	916.7 ± 388.6	1000.0 ± 468.9
Insulin glargine U100 [U/kg]	0.09 ± 0.05	0.12 ± 0.09

Values are presented as mean ± standard deviation (SD). FMD, flow-mediated dilation; HbA1c, glycated hemoglobin; FPG, fasting plasma glucose; GA, glycated albumin; BMI, body mass index; Cr, serum creatinine; eGFR, estimated glomerular filtration rate; UA, uric acid; LDL-C, low-density lipoprotein cholesterol; HDL-C, high-density lipoprotein cholesterol; TG, triglycerides; sBP, systolic blood pressure; dBP, diastolic blood pressure.

### Endothelial function

The average baseline FMD values before and after 12-weeks of treatment with empagliflozin or glimepiride are presented in Table 2. No significant difference was observed in FMD changes between the empagliflozin and glimepiride groups. Results were similar for both FAS and PPS (S1 Table).

**Table 2:** FMD (%) with treatment for full analysis set by mixed model analysis.

	Empagliflozin group (*n* = 33)			Glimepiride group (*n* = 30)			Difference (Empagliflozin-Glimepiride)		
	mean	95%CI	P-value vs. baseline	mean	95%CI	P-value vs. baseline	mean	95%CI	P-value
FMD(0)	5.38	4.67, 6.10	-	5.42	4.67, 6.17	-	-0.04	-1.08, 1.00	0.937
FMD(12)	5.27	4.52, 6.02	0.810	5.08	4.31, 5.86	0.474	0.19	-0.89, 1.27	0.729
ΔFMD	-0.11	-1.02, 0.80	-	-0.34	-1.28, 0.60	-	0.23	-1.08, 1.54	0.726

Values are presented as least square means (LS mean) and 95% confidence interval (95%CI). P-values represent the results of linear mixed model analysis with outcome as the dependent variable in FAS. In the model, subjects were the random factors, and time (Baseline or Week 12) and group (Empagliflozin or Glimepiride) and their interaction terms were fixed factors. FAS analysis. FMD, flow-mediated dilation; FMD (0) means the baseline FMD value at 0 week. FMD (12) means the FMD value at 12 weeks after additional treatment; Δ indicates the change in the FMD value between 0 and 12 weeks.

In both subgroups that exhibited a lower than median baseline FMD, theΔFMD did not significantly differ between the two groups (*P* = 0.84; Table 3).

**Table 3:** Change in FMD (% ± SD) after additional treatment in subgroups with lower than median baseline FMD.

	Empagliflozin group (*n* = 17)			Glimepiride group (*n* = 17)			Difference (Empagliflozin-Glimepiride)		
	LS mean	95%CI	P-value vs. baseline	LS mean	95%CI	P-value vs. baseline	LS mean	95%CI	P-value
FMD(0)	3.93	3.14, 4.72	-	4.16	3.38, 4.95	-	-0.24	-1.35, 0.88	0.673
FMD(12)	4.54	3.68, 5.40	0.223	4.92	4.09, 5.75	0.126	-0.38	-1.57, 0.82	0.529
ΔFMD	0.61	-0.39, 1.61	-	0.75	-0.22, 1.73	-	-0.14	-1.54, 1.25	0.836

Values are presented as least square means (LS mean) and 95% confidence interval (95%CI). P-values represent the results of linear mixed model analysis with outcome as the dependent variable in FAS. In the model, subjects were the random factors, and time (Baseline or Week 12) and group (Empagliflozin or Glimepiride) and their interaction terms were fixed factors. FMD, flow-mediated dilation; FMD (0) means the baseline FMD value at 0 week. FMD (12) means the FMD value at 12 weeks after additional treatment; Δ indicates the change in the FMD value between 0 and 12 weeks.

### Metabolic markers

The fasting plasma glucose (FPG) levels remained unchanged in both groups, with no significant difference evident between the two groups following treatment (*P* = 0.52; Table 4). HbA1c and glycated albumin (GA) levels significantly decreased in both groups (*P* ≤ 0.05). However, the changes in these metabolic markers were not significantly different between the two groups (ΔHbA1c, *P* = 0.82 and ΔGA, *P* = 0.4). Uric acid (UA) was significantly decreased in the empagliflozin group (*P* < 0.001). A significant difference between the two groups was also noted in the ΔUA (*P* < 0.001). Systolic blood pressure (*P* = 0.61) and diastolic blood pressure (*P* = 0.71) did not change significantly between the two groups. Empagliflozin treatment significantly increased low-density lipoprotein-C (LDL-C; *P* = 0.002). In contrast, triglycerides (TG; *P* = 0.46) and high-density lipoprotein-C (HDL-C; *P* = 0.65) did not change significantly between the two groups. These results were similar for both FAS and PPS (S2 Table).

**Table 4:** Metabolic markers with treatment for full analysis set by mixed model analysis.

	Empagliflozin group (*n* = 33)			Glimepiride group (*n* = 30)			Difference		
							(Empagliflozin-Glimepiride)		
	LS mean	95%CI	P-value vs. baseline	LS mean	95%CI	P-value vs. baseline	LS mean	95%CI	P-value
FPG (mg/dL)									
Baseline	143.52	124.13, 162.90	-	125.60	105.27, 145.93	-	17.92	-10.18, 46.01	0.208
Week 12	128.68	108.73, 148.62	0.096	118.91	98.16, 139.67	0.465	9.76	-19.02, 38.55	0.502
ΔFPG	-14.84	-32.39, 2.71	-	-6.69	-24.90, 11.53	-	-8.15	-33.45, 17.14	0.521
HbA1c (%)									
Baseline	7.04	6.69, 7.38	-	6.64	6.28, 7.00	-	0.40	-0.10, 0.90	0.116
Week 12	6.81	6.46, 7.16	0.005	6.38	6.02, 6.75	0.003	0.43	-0.08, 0.93	0.097
ΔHbA1c	-0.23	-0.39, -0.07	-	-0.26	-0.42, -0.09	-	0.03	-0.20, 0.25	0.819
GA (%)									
Baseline	17.16	15.99, 18.34	-	16.33	15.12, 17.54	-	0.83	-0.85, 2.52	0.328
Week 12	16.18	15.00, 17.36	<0.001	15.68	14.46, 16.90	0.024	0.50	-1.20, 2.20	0.560
ΔGA	-0.99	-1.53, -0.44	-	-0.65	-1.22, -0.09	-	-0.33	-1.12, 0.45	0.396
Cr (mg/dL)									
Baseline	0.76	0.71, 0.82	-	0.74	0.68, 0.80	-	0.02	-0.06, 0.10	0.614
Week 12	0.79	0.73, 0.84	0.050	0.75	0.69, 0.80	0.831	0.04	-0.04, 0.12	0.322
ΔCr	0.02	0.00, 0.04	-	0.00	-0.02, 0.03	-	0.02	-0.01, 0.05	0.222
eGFR (mL min^−1^ 1.73 m^−2^)									
Baseline	75.62	70.02, 81.23	-	81.52	75.64, 87.40	-	-5.90	-14.02, 2.22	0.152
Week 12	72.67	67.02, 78.33	0.038	81.13	75.21, 87.05	0.787	-8.46	-16.65, -0.27	0.043
ΔeGFR	-2.95	-5.72, -0.17	-	-0.39	-3.27, 2.49	-	-2.56	-6.56, 1.45	0.206
UA (mg/dL)										
Baseline	5.44	4.99, 5.88	-	5.42	4.95, 5.89	-	0.02	-0.63, 0.67	0.947
Week 12	4.81	4.35, 5.26	<0.001	5.68	5.20, 6.15	0.065	-0.87	-1.53, -0.22	0.010
ΔUA	-0.63	-0.90, -0.36	-	0.26	-0.02, 0.54	-	-0.89	-1.28, -0.51	<0.001
Body weight(kg)								
Baseline	69.86	64.71, 75.02	-	69.19	63.78, 74.59	-	0.68	-6.79, 8.14	0.857
Week 12	69.28	64.12, 74.44	0.255	70.39	64.98, 75.80	0.026	-1.11	-8.59, 6.37	0.767
ΔBW	-0.58	-1.60, 0.43	-	1.20	0.15, 2.26	-	-1.79	-3.25, -0.32	0.018
Waist circumference(cm)									
Baseline	91.82	87.31, 96.33	-	90.59	86.01, 95.17	-	1.22	-5.20, 7.65	0.705
Week 12	91.20	86.69, 95.70	0.183	91.75	87.17, 96.33	0.015	-0.56	-6.98, 5.87	0.863
Δwaist	-0.62	-1.54, 0.30	-	1.16	0.24, 2.08	-	-1.78	-3.09, -0.48	0.008
Blood pressure (mmHg)								
sBP									
Baseline	129.59	123.44, 135.74	-	130.90	124.50, 137.30	-	-1.31	-10.19, 7.56	0.769
Week 12	129.93	123.68, 136.18	0.891	129.39	122.87, 135.90	0.558	0.55	-8.48, 9.57	0.905
ΔsBP	0.35	-4.68, 5.38	-	-1.51	-6.66, 3.63	-	1.86	-5.34, 9.06	0.607
dBP									
Baseline	80.35	76.76, 83.94	-	78.63	74.91, 82.36	-	1.71	-3.46, 6.89	0.512
Week 12	79.67	75.99, 83.35	0.717	78.94	75.12, 82.76	0.873	0.72	-4.58, 6.03	0.787
ΔdBP	-0.68	-4.43, 3.07	-	0.31	-3.54, 4.16	-	-0.99	-6.37, 4.39	0.713
LDL-C (mg/dL)									
Baseline	95.73	85.69, 105.77	-	89.47	78.93, 100.00	-	6.26	-8.29, 20.81	0.394
Week 12	108.72	98.44, 119.00	0.002	94.10	83.38, 104.81	0.273	14.62	-0.22, 29.47	0.053
ΔLDL-C	12.99	4.92, 21.07	-	4.63	-3.74, 13.00	-	8.36	-3.27, 19.99	0.156
HDL-C (mg/dL)								
Baseline	56.97	51.65, 62.29	-	58.07	52.48, 63.65	-	-1.10	-8.81, 6.62	0.778
Week 12	57.82	52.40, 63.25	0.648	56.85	51.20, 62.51	0.531	0.97	-6.86, 8.81	0.806
ΔHDL-C	0.85	-2.87, 4.58	-	-1.21	-5.07, 2.65	-	2.07	-3.30, 7.43	0.443
TG (mg/dL)									
Baseline	186.88	148.53, 225.23	-	171.37	131.14, 211.59	-	15.51	-40.06, 71.09	0.581
Week 12	171.85	132.08, 211.61	0.462	183.40	142.12, 224.69	0.570	-11.56	-68.88, 45.76	0.690
ΔTG	-15.03	-55.62, 25.56	-	12.04	-30.12, 54.20	-	-27.07	-85.59, 31.45	0.358
Body fluid volume (L)								
Baseline	35.80	33.06, 38.53	-	36.63	33.81, 39.45	-	-0.83	-4.77, 3.10	0.673
Week 12	35.48	32.74, 38.21	0.238	36.27	33.45, 39.09	0.185	-0.79	-4.72, 3.14	0.688
ΔBody fluid	-0.32	-0.85, 0.22	-	-0.36	-0.89, 0.18	-	0.04	-0.72, 0.80	0.916
Total fat mass (kg)									
Baseline	21.40	18.04, 24.76	-	19.93	16.47, 23.40	-	1.47	-3.36, 6.30	0.545
Week 12	20.83	17.48, 24.19	0.192	21.12	17.66, 24.59	0.008	-0.29	-5.11, 4.54	0.906
Δtotal fat mass	-0.57	-1.43, 0.29	-	1.19	0.33, 2.05	-	-1.76	-2.97, -0.54	0.006
Insulin glargine U100 (U)								
Baseline	9.03	6.58, 11.48	-	11.70	9.13, 14.27	-	-2.67	-6.22, 0.89	0.139
Week 12	8.03	5.56, 10.51	0.071	9.59	7.01, 12.18	<0.001	-1.56	-5.14, 2.02	0.387
Δinsulin glargine	-1.00	-2.08, 0.09	-	-2.11	-3.23, -0.98	-	1.11	-0.46, 2.67	0.162

Values are presented as least square means (LS mean) and 95% confidence interval (95%CI). P-values represent the results of linear mixed model analysis with outcome as the dependent variable in FAS. In the model, subjects were the random factors, and time (Baseline or Week 12) and group (Empagliflozin or Glimepiride) and their interaction terms were fixed factors. Δ indicates the changes in the metabolic markers between 0 and 12 weeks. HbA1c, glycated hemoglobin; GA, glycated albumin; Cr, serum creatinine; eGFR, estimated glomerular filtration rate; UA, uric acid; sBP, systolic blood pressure; dBP, diastolic blood pressure; LDL-C, low-density lipoprotein cholesterol; HDL-C, high-density lipoprotein cholesterol; TG, triglycerides.

A significant increase in body weight (*P* < 0.05) was observed after glimepiride treatment. In addition, a significant difference was observed in the change in body weight between the two groups (*P* = 0.02). However, in the subgroup of patients for whom a decrease in body weight was observed, ΔFMD was not significantly different between the two groups (*P* = 0.62; Table 5). Glimepiride treatment also induced a significant increase in waist circumference (*P* = 0.02). Moreover, a significant difference was observed in the change in waist circumference between the two groups (*P* = 0.008). With regard to body composition, glimepiride treatment led to a significant increase in total fat mass (*P* = 0.008). In comparison, empagliflozin significantly decreased body fluid volume (*P* = 0.03) only in PPS (S2 Table).

**Table 5:** Changes in FMD (% ± SD) in patient subgroup with decreased body weight during observation period.

	Empagliflozin group (*n* = 20)			Glimepiride group (*n* = 10)			Difference (Empagliflozin-Glimepiride)		
	LS mean	95%CI	P-value vs. baseline	LS mean	95%CI	P-value vs. baseline	LS mean	95%CI	P-value
FMD(0)	5.29	4.34, 6.23	-	4.22	2.89, 5.55	-	1.06	-0.57, 2.70	0.196
FMD(12)	5.80	4.79, 6.81	0.366	5.05	3.58, 6.52	0.318	0.75	-1.03, 2.54	0.399
ΔFMD	0.52	-0.64, 1.67	-	0.83	-0.84, 2.50	-	0.31	-1.72, 2.34	0.756

Values are presented as least square means (LS mean) and 95% confidence interval (95%CI). P-values represent the results of linear mixed model analysis with outcome as the dependent variable in FAS. In the model, subjects were the random factors, and time (Baseline or Week 12) and group (Empagliflozin or Glimepiride) and their interaction terms were fixed factors.

FMD, flow-mediated dilation; Δ indicates the change in FMD value between 0 and 12 weeks.

The fasting C-peptide immunoreactivity levels (CPR; *P* = 0.57), homeostasis model assessment 2 steady state beta cell (%B) function (*P* = 0.29), homeostasis model assessment 2 insulin sensitivity (%S) (*P* = 0.42), and homeostasis model assessment 2 insulin resistance (*P* = 0.51) were not significantly different between the two groups (S3 Table). These results were similar for both FAS and PPS (S4 Table). In addition, empagliflozin and glimepiride did not affect insulin secretion, insulin sensitivity, pancreatic β-cell function, or insulin resistance. Few arteriosclerosis markers were correlated with ΔFMD; these markers included body weight changes, ΔHbA1c, ΔHDL-C, and ΔTG. Note, ΔFMD would have been greater if the baseline FMD was lower (S5 Table).

## Discussion

In the present study, we assessed the effects of empagliflozin and glimepiride, a commonly prescribed sulfonylurea, on endothelial function in patients with type 2 diabetes using FMD. Fasting plasma glucose (FPG), HbA1c, and GA were equally improved in both the empagliflozin and glimepiride groups; however, there was no significant change in FMD in either of the groups. The average difference between the two groups of FMD was small and 95% confidence interval was similar to the reproducibility of FMD [20]. The glimepiride result is in line with a previous report where glimepiride treatment did not result in improved endothelial function [19]. In addition, empagliflozin had no effect on endothelial function, irrespective of the improvement in glucose levels.

This study was conducted in patients in a glucose-controlled state to observe changes in endothelial function induced by drugs that lack the ability to improve glucose levels, and to exclude the effects of glucotoxicity and temporary endothelial dysfunction associated with high blood glucose. The baseline HbA1c was targeted in this study; however, severe hypoglycemia did not occur after reducing the insulin glargine U100 dose, and the fasting plasma glucose was maintained under 90 mg/dL. The insulin dose was also decreased significantly in both groups, which may have caused the observed decrease in body weight. This significant change was confirmed only in PPS. HbA1c was significantly reduced in the empagliflozin group; however, this change did not affect FMD. In contrast, a correlation between decreased visceral adipose tissue mass, waist circumference, and ΔFMD has been reported in previous studies [8, 21]. Our findings suggest that empagliflozin had no effect on FMD, although empagliflozin did improve HbA1c.

Long-term administration of empagliflozin maintains a stable eGFR [22]; however, we observed a deterioration in renal function in the empagliflozin group during our relatively short observation period. Empagliflozin decreases hyperfiltration induced by diuresis in the early stages of treatment [23]. This may account for the observed changes in renal dynamics induced by empagliflozin. Canagliflozin reduces the risk of kidney failure at a median follow-up time of 2.62 years [24]. In addition, while losartan treatment results in a rapid initial decline in renal function, the decrease in long-term renal function is slowed [25]. Therefore, empagliflozin could prevent the progression of kidney disease over an extended period. However, an initial rapid decline in renal function could result in kidney failure [26].

The clinical effects of empagliflozin on the lipid composition is unclear; however, empagliflozin marginally increased LDL-C levels [27]. This is in contradiction with the coronary-protective effect of empagliflozin. We observed that empagliflozin significantly increased the levels of LDL-C. It is possible that the reduction in volume of body fluids [28] and the loss of calories [29] contributed to this change. However, the underlying mechanism is unclear and therefore, further research is needed.

InBody720, an eight-polar BIA, accurately estimates the total and appendicular body composition, independent of age and sex [18]. In our study, body fluid volume was significantly decreased following 12 weeks of empagliflozin treatment. This change was confirmed only in PPS and it was unclear whether the effect of empagliflozin on the volume of body fluids was greater than that of glimepiride; however, there is a tendency of a greater decreases in the fluid volume in response to empagliflozin, compared to that against glimepiride, with less individual variations. The empagliflozin group may have a clear reduction effect on the fluid volume. Empagliflozin improves hospitalization rates after heart failure [30] and it possibly attenuates the effects of this disease by decreasing body fluid volume. During the observational period of our study, heart failure was not observed in either of the treatment groups. Thus, empagliflozin might have a coronary-protective effect independent of its impact on endothelial function.

Empagliflozin decreases congestive heart failure through its diuretic activities; however, its effect on reducing cardiovascular event risk is unclear. Compared with loop diuretics, SGLT2 inhibitors produce a greater reduction in interstitial fluid volume relative to blood volume [31]. It is hypothesized that improvements in systemic congestion and renal function, derived from decreased hyperfiltration, result in the prevention of cardiovascular events without reducing arterial filling and perfusion.

An increase in FMD may be achieved in circumstances where the baseline FMD is low [32]. In addition, baseline FMD is related to HbA1c [6]. If baseline HbA1c levels were higher, baseline FMD would be lower and endpoint FMD would improve. However, in both subgroups that showed a lower than median baseline FMD, the ΔFMD did not significantly differ between the two groups (*P* = 0.83; Table 3).

SGLT2 inhibitors have a secondary preventive effect on adverse cardiovascular events; however, they lack a primary preventive effect [12]. The results from the DECLARE-TIMI 58 study demonstrated that dapagliflozin has both primary, as well as secondary, effects on preventing adverse cardiovascular events. Dapagliflozin decreases cardiovascular death and hospitalization due to heart failure in patients with no history of cardiovascular disease [33]. However, in our study, patients did not present similar backgrounds, and hence, it was necessary to undertake a secondary intervention.

The effects of other hyperglycemic agents, such as pioglitazone and glucagon-like peptide 1 (GLP-1) analogs, on improving endothelial function have been reported previously [19, 34, 35]. A dipeptidyl peptidase 4 (DPP-4) inhibitor either improves [32, 36], has no effect [37], or worsens [38] endothelial function, but does not affect cardiovascular events [39, 40]. GLP-1 analog treatment enhances [35, 41], or has no effect on [42], endothelial function. In patients with type 2 diabetes, liraglutide, a GLP-1 analog, successfully prevents nonfatal myocardial infarction or stroke, and death from cardiovascular causes [43]. In line with these diverse findings, results from a meta-analysis showed that significant heterogeneity existed between DPP-4 inhibitors and GLP-1 [44]. Factors such as the size of the study, duration of intervention, and age or sex of the participants did not affect the mean difference in FMD [44]. However, FMD change was dependent on baseline FMD values. Therefore, the low baseline FMD in our study compared to that in other studies [32, 36] could have affected our results.

Several limitations were evident in this study. First, as the study participants were outpatients, we were unable to exclude the possibility that they had smoked or eaten before the FMD examination. Moreover, non-compliance of dietary requirements in some patients might have affected the observed change in body weight. Additionally, we had included wash-out periods of anti-diabetic therapy for 12 weeks before the study; there were no significant differences between the drugs (S6 Table); however, long-term effects of anti-diabetic therapy before admission cannot be completely ruled out. The fasting blood glucose did not improve significantly in both the groups, because the baseline HbA1c was controlled. The effects of higher doses of glimepiride and empagliflozin in improving the fasting blood glucose should be considered. Finally, although the number of patients included was greater than that required to detect a significant difference in a comparison of the two groups, the observation period was relatively short. The monitoring period required for FMD changes to be detectable has not been established; however, a longer observation period is nonetheless required for future studies to monitor any adverse events.

## Conclusions

We found that empagliflozin did not improve endothelial function compared to glimepiride in patients with type 2 diabetes without previous cardiovascular disease over a 12-week treatment period. However, empagliflozin may have a clear effect on the reduction of fluid volume. Thus, the coronary-protective effect of empagliflozin might not be derived from its ability to prevent endothelial dysfunction, but rather from a reduced risk of heart failure.

## Supporting information

S1 Fig Study flow chart. Patients who took metformin and insulin glargine U100 were randomized to the empagliflozin or glimepiride groups. Flow-mediated dilation (FMD), blood examination, body weight, and blood pressure were assessed at study baseline and endpoints.
(TIF)

S1 Table FMD (% ± SD) with treatment for per protocol set. (DOCX)

S2 Table Changes in metabolic markers for per protocol set. (DOCX)

S3 Table Changes in metabolic markers for full analysis set. (DOCX)

S4 Table Changes in metabolic markers for per protocol set. (DOCX)

S5 Table Associations between arterial sclerosis markers and ΔFMD for per protocol set (n = 58). (DOCX)

S6 Table Association between anti-diabetic therapy and ΔFMD in the empagliflozin and glimepiride groups for per protocol set. (DOCX)

S1 Protocol Protocol in original language. (DOC)

S2 Protocol Protocol translated into English. (DOC)

S1 Checklist CONSORT 2010 checklist. (DOC)

S1 Dataset All raw dataset. (XLSX)

## References

[1] Schachinger V, Britten MB, Zeiher AM. Prognostic impact of coronary vasodilator dysfunction on adverse long-term outcome of coronary heart disease. Circulation. 2000; 101: 1899–1906. doi: 10.1161/01.cir.101.16.1899 10779454

[2] Corretti MC, Anderson TJ, Benjamin EJ, Celermajer D, Charbonneau F, Creager MA, et al. Guidelines for the ultrasound assessment of endothelial-dependent flow-mediated vasodilation of the brachial artery: a report of the International Brachial Artery Reactivity Task Force. J Am Coll Cardiol. 2002; 39: 257–265. doi: 10.1016/s0735-1097(01)01746-6 11788217

[3] Yeboah J, Folsom AR, Burke GL, Johnson C, Polak JF, Post W, et al. Predictive value of brachial flow-mediated dilation for incident cardiovascular events in a population-based study: the multi-ethnic study of atherosclerosis. Circulation. 2009; 120: 502–509. doi: 10.1161/CIRCULATIONAHA.109.864801 19635967 PMC2740975

[4] Ter Avest E, Stalenhoef AF, de Graaf J. What is the role of non-invasive measurements of atherosclerosis in individual cardiovascular risk prediction? Clin Sci. 2007; 112:507–516. 17419684

[5] Deanfield JE, Halcox JP, Rabelink TJ. Endothelial function and dysfunction: testing and clinical relevance. Circulation. 2007; 115:1285–1295. doi: 10.1161/CIRCULATIONAHA.106.652859 17353456

[6] Maruhashi T, Soga J, Fujimura N, Idei N, Mikami S, Iwamoto Y, et al. Relationship between flow-mediated vasodilation and cardiovascular risk factors in a large community-based study. Heart. 2013; 99:1837–1842. doi: 10.1136/heartjnl-2013-304739 24153417 PMC3841746

[7] Benjamin EJ, Larson MG, Keyes MJ, Mitchell GF, Vasan RS, Keaney JF, et al. Clinical correlates and heritability of flow-mediated dilation in the community: the Framingham Heart Study. Circulation. 2004; 109:613–619. doi: 10.1161/01.CIR.0000112565.60887.1E 14769683

[8] Rittig K, Hieronimus A, Thamer C, Machann J, Peter A, Stock J, et al. Reducing visceral adipose tissue mass is essential for improving endothelial function in type 2 diabetes prone individuals. Atherosclerosis. 2010; 212:575–579. doi: 10.1016/j.atherosclerosis.2010.06.042 20667538

[9] Kadowaki T, Haneda M, Inagaki N, Terauchi Y, Taniguchi A, Koiwai K, et al. Efficacy and safety of empagliflozin monotherapy for 52 weeks in Japanese patients with type 2 diabetes: a randomized, double-blind, parallel-group study. Advances in therapy. 2015; 32: 306–318. doi: 10.1007/s12325-015-0198-0 25845768

[10] Roden M, Merker L, Christiansen AV, Roux F, Salsali A, Kim G, et al. Safety, tolerability and effects on cardiometabolic risk factors of empagliflozin monotherapy in drug-naive patients with type 2 diabetes: a double-blind extension of a Phase III randomized controlled trial. Cardiovasc Diabetol. 2015; 14: 154. doi: 10.1186/s12933-015-0314-0 26701110 PMC4690334

[11] Zinman B, Wanner C, Lachin JM, Fitchett D, Bluhmki E, Hantel S, et al. Empagliflozin, Cardiovascular Outcomes, and Mortality in Type 2 Diabetes. N Engl J Med. 2015; 373: 2117–2128. doi: 10.1056/NEJMoa1504720 26378978

[12] Zelniker TA, Wiviott SD, Raz I, Im K, Goodrich EL, Bonaca MP, et al. SGLT2 inhibitors for primary and secondary prevention of cardiovascular and renal outcomes in type 2 diabetes: a systematic review and meta-analysis of cardiovascular outcome trials. Lancet. 2019; 393: 31–39. doi: 10.1016/S0140-6736(18)32590-X 30424892

[13] UK Prospective Diabetes Study (UKPDS) Group. Intensive blood-glucose control with sulphonylureas or insulin compared with conventional treatment and risk of complications in patients with type 2 diabetes (UKPDS 33). UK Prospective Diabetes Study (UKPDS) Group. Lancet. 1998; 352: 837–853. 9742976

[14] Rao AD, Kuhadiya N, Reynolds K, Fonseca VA. Is the combination of sulfonylureas and metformin associated with an increased risk of cardiovascular disease or all-cause mortality?: a meta-analysis of observational studies. Diabetes Care. 2008; 31: 1672–1678. doi: 10.2337/dc08-0167 18458139 PMC2494623

[15] Haring HU, Merker L, Seewaldt-Becker E, Weimer M, Meinicke T, Woerle HJ, et al. Empagliflozin as add-on to metformin plus sulfonylurea in patients with type 2 diabetes: a 24-week, randomized, double-blind, placebo-controlled trial. Diabetes Care. 2013; 36: 3396–3404. doi: 10.2337/dc12-2673 23963895 PMC3816918

[16] Haring HU, Merker L, Seewaldt-Becker E, Weimer M, Meinicke T, Broedl UC, et al. Empagliflozin as add-on to metformin in patients with type 2 diabetes: a 24-week, randomized, double-blind, placebo-controlled trial. Diabetes Care. 2014; 37: 1650–1659. doi: 10.2337/dc13-2105 24722494

[17] Urakaze M, Yamazaki K, Usui I, Iwata M, Uno T, Murakami S, et al. Glimepiride (0.5 mg/day) Administration Improves Glycemic Control without Weight Gain in Japanese Type 2 Diabetic Patients. J. Japan DiabSoc. 2007; 50: 835–841.

[18] Malavolti M, Mussi C, Poli M, Fantuzzi AL, Salvioli G, Battistini N, et al. Cross-calibration of eight-polar bioelectrical impedance analysis versus dual-energy X-ray absorptiometry for the assessment of total and appendicular body composition in healthy subjects aged 21–82 years. Ann hum biol. 2003; 30: 380–391. doi: 10.1080/0301446031000095211 12881138

[19] Papathanassiou K, Naka KK, Kazakos N, Kanioglou C, Makriyiannis D, Pappas K, et al. Pioglitazone vs glimepiride: Differential effects on vascular endothelial function in patients with type 2 diabetes. Atherosclerosis. 2009; 205: 221–226. doi: 10.1016/j.atherosclerosis.2008.11.027 19135671

[20] Charakida M, de Groot E, Loukogeorgakis SP, Khan T, Lüscher T, Kastelein JJ, et al. Variability and reproducibility of flow-mediated dilatation in a multicentre clinical trial. Eur Heart J. 2013; 34: 3501–3507. doi: 10.1093/eurheartj/eht223 23821401

[21] Miyazaki S, Hiasa Y, Takahashi T, Tobetto Y, Chen H, Mahara K, et al. Waist circumference reduction is more strongly correlated with the improvement in endothelial function after acute coronary syndrome than body mass index reduction. J Cardiol. 2010; 55: 266–273. doi: 10.1016/j.jjcc.2009.11.006 20206081

[22] Wanner C, Inzucchi SE, Lachin JM, Fitchett D, von Eynatten M, Mattheus M, et al. Empagliflozin and Progression of Kidney Disease in Type 2 Diabetes. N Engl J Med. 2016; 375: 323–334. doi: 10.1056/NEJMoa1515920 27299675

[23] Vallon V, Gerasimova M, Rose MA, Masuda T, Satriano J, Mayoux E, et al. SGLT2 inhibitor empagliflozin reduces renal growth and albuminuria in proportion to hyperglycemia and prevents glomerular hyperfiltration in diabetic Akita mice. Am J Physiol Ren Physiol. 2014; 306: F194–204. doi: 10.1152/ajprenal.00520.2013 24226524 PMC3920018

[24] Perkovic V, Jardine MJ, Neal B, Bompoint S, Heerspink HJL, Charytan DM, et al. Canagliflozin and Renal Outcomes in Type 2 Diabetes and Nephropathy. N Engl J Med. 2019; 380: 2295–2306. doi: 10.1056/NEJMoa1811744 30990260

[25] Holtkamp FA, de Zeeuw D, Thomas MC, Cooper ME, de Graeff PA, Hillege HJ, et al. An acute fall in estimated glomerular filtration rate during treatment with losartan predicts a slower decrease in long-term renal function. Kidney Int. 2011; 80: 282–287. doi: 10.1038/ki.2011.79 21451458

[26] Levey AS, Coresh J. Chronic kidney disease. Lancet. 2012; 379: 165–180. doi: 10.1016/S0140-6736(11)60178-5 21840587

[27] Ferrannini E, DeFronzo RA. Impact of glucose-lowering drugs on cardiovascular disease in type 2 diabetes. Eur Heart J. 2015; 36: 2288–2296. doi: 10.1093/eurheartj/ehv239 26063450

[28] Feingold KR, Anawalt B, Boyce A, Chrousos G, de Herder WW, Dungan K, et al. Role of Glucose and Lipids in the Atherosclerotic Cardiovascular Disease of Patients with Diabetes. Endotext. 2000–2020. 25905182

[29] Inagaki N, Goda M, Yokota S, Maruyama N, Iijima H. Effects of Baseline Blood Pressure and Low-Density Lipoprotein Cholesterol on Safety and Efficacy of Canagliflozin in Japanese Patients with Type 2 Diabetes Mellitus. Advances in therapy. 2015; 32: 1085–1103. doi: 10.1007/s12325-015-0255-8 26530268 PMC4662712

[30] Fitchett D, Zinman B, Wanner C, Lachin JM, Hantel S, Salsali A, et al. Heart failure outcomes with empagliflozin in patients with type 2 diabetes at high cardiovascular risk: results of the EMPA-REG OUTCOME trial. Eur Heart J. 2016; 37: 1526–1534. doi: 10.1093/eurheartj/ehv728 26819227 PMC4872285

[31] Hallow KM, Helmlinger G, Greasley PJ, McMurray JJV, Boulton DW. Why do SGLT2 inhibitors reduce heart failure hospitalization? A differential volume regulation hypothesis. Diabetes Obes Metab. 2018; 20: 479–487. doi: 10.1111/dom.13126 29024278

[32] Shigiyama F, Kumashiro N, Miyagi M, Iga R, Kobayashi Y, Kanda E, et al. Linagliptin improves endothelial function in patients with type 2 diabetes: A randomized study of linagliptin effectiveness on endothelial function. J Diabetes Investig. 2017; 8: 330–340. doi: 10.1111/jdi.12587 27868359 PMC5415473

[33] Wiviott SD, Raz I, Bonaca MP, Mosenzon O, Kato ET, Cahn A, et al. Dapagliflozin and Cardiovascular Outcomes in Type 2 Diabetes. N Engl J Med. 2019; 380: 347–357. doi: 10.1056/NEJMoa1812389 30415602

[34] Tsuchiya K, Akaza I, Yoshimoto T, Hirata Y. Pioglitazone improves endothelial function with increased adiponectin and high-density lipoprotein cholesterol levels in type 2 diabetes. Endocr J. 2009; 56: 691–698. doi: 10.1507/endocrj.k08e-308 19506330

[35] Lambadiari V, Pavlidis G, Kousathana F, Varoudi M, Vlastos D, Maratou E, et al. Effects of 6-month treatment with the glucagon like peptide-1 analogue liraglutide on arterial stiffness, left ventricular myocardial deformation and oxidative stress in subjects with newly diagnosed type 2 diabetes. Cardiovasc Diabetol. 2018; 17: 8. doi: 10.1186/s12933-017-0646-z 29310645 PMC5759220

[36] Nakamura K, Oe H, Kihara H, Shimada K, Fukuda S, Watanabe K, et al. DPP-4 inhibitor and alpha-glucosidase inhibitor equally improve endothelial function in patients with type 2 diabetes: EDGE study. Cardiovasc Diabetol. 2014; 13: 110. doi: 10.1186/s12933-014-0110-2 25074318 PMC4149239

[37] Nomoto H, Miyoshi H, Furumoto T, Oba K, Tsutsui H, Inoue A, et al. A Randomized Controlled Trial Comparing the Effects of Sitagliptin and Glimepiride on Endothelial Function and Metabolic Parameters: Sapporo Athero-Incretin Study 1 (SAIS1). PLoS One. 2016; 11: e0164255. doi: 10.1371/journal.pone.0164255 27711199 PMC5053511

[38] Ayaori M, Iwakami N, Uto-Kondo H, Sato H, Sasaki M, Komatsu T, et al. Dipeptidyl peptidase-4 inhibitors attenuate endothelial function as evaluated by flow-mediated vasodilatation in type 2 diabetic patients. J Am Heart Assoc. 2013; 2: e003277. doi: 10.1161/JAHA.112.003277 23525426 PMC3603233

[39] Green JB, Bethel MA, Armstrong PW, Buse JB, Engel SS, Garg J, et al. Effect of Sitagliptin on Cardiovascular Outcomes in Type 2 Diabetes. N Engl J Med. 2015; 373: 232–242. doi: 10.1056/NEJMoa1501352 26052984

[40] Scirica BM, Bhatt DL, Braunwald E, Steg PG, Davidson J, Hirshberg B, et al. Saxagliptin and cardiovascular outcomes in patients with type 2 diabetes mellitus. N Engl J Med. 2013; 369: 1317–1326. doi: 10.1056/NEJMoa1307684 23992601

[41] Ceriello A, Esposito K, Testa R, Bonfigli AR, Marra M, Giugliano D. The possible protective role of glucagon-like peptide 1 on endothelium during the meal and evidence for an "endothelial resistance" to glucagon-like peptide 1 in diabetes. Diabetes Care. 2011; 34: 697–702. doi: 10.2337/dc10-1949 21273492 PMC3041210

[42] Nomoto H, Miyoshi H, Furumoto T, Oba K, Tsutsui H, Miyoshi A, et al. A Comparison of the Effects of the GLP-1 Analogue Liraglutide and Insulin Glargine on Endothelial Function and Metabolic Parameters: A Randomized, Controlled Trial Sapporo Athero-Incretin Study 2 (SAIS2). PLoS One. 2015; 10: e0135854. doi: 10.1371/journal.pone.0135854 26284918 PMC4540491

[43] Marso SP, Daniels GH, Brown-Frandsen K, Kristensen P, Mann JF, Nauck MA, et al. Liraglutide and Cardiovascular Outcomes in Type 2 Diabetes. N Engl J Med. 2016; 375: 311–322. doi: 10.1056/NEJMoa1603827 27295427 PMC4985288

[44] Batzias K, Antonopoulos AS, Oikonomou E, Siasos G, Bletsa E, Stampouloglou PK, et al. Effects of Newer Antidiabetic Drugs on Endothelial Function and Arterial Stiffness: A Systematic Review and Meta-Analysis. J Diabetes Res. 2018; 2018: 1232583. doi: 10.1155/2018/1232583 30622967 PMC6304901

