# Supplementary figures and images for: Comparison of the effects of empagliflozin and glimepiride on endothelial function in patients with type 2 diabetes: A randomized controlled study
Source: PLoS One. 2022 Feb 16;17(2):e0262831. doi: 10.1371/journal.pone.0262831 (PMC8849516; doi:10.1371/journal.pone.0262831)

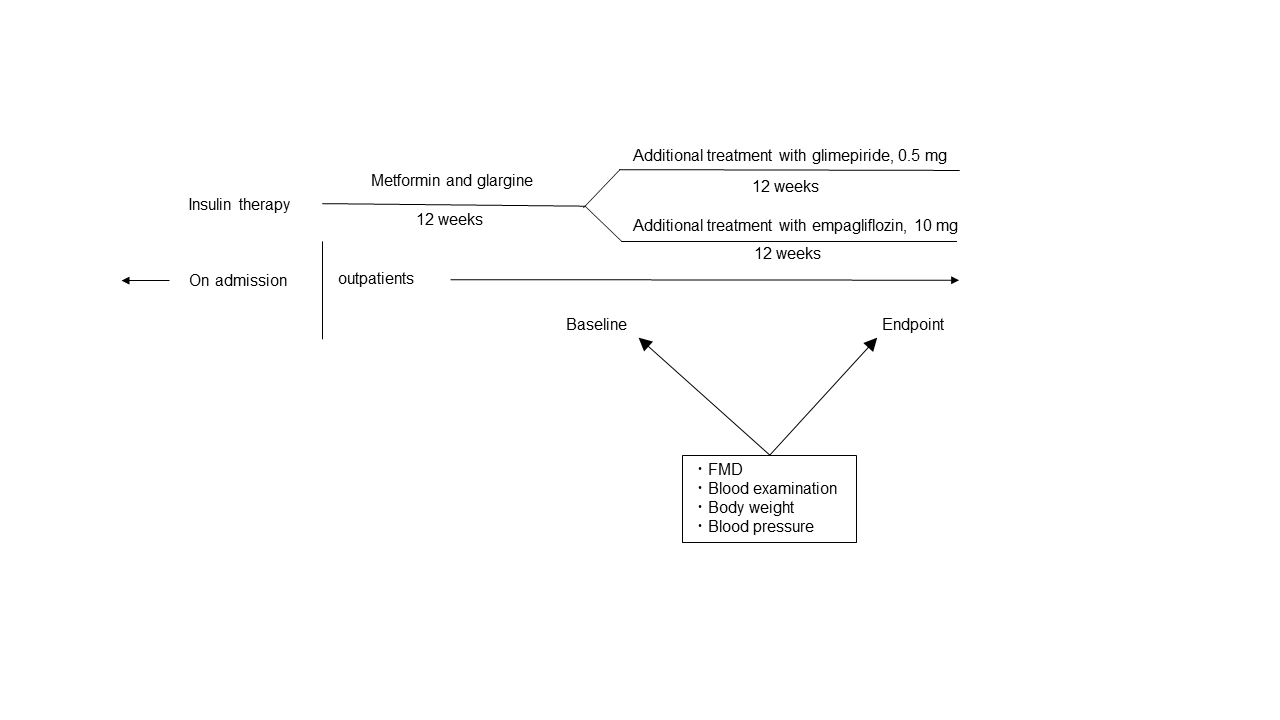

Supplement: S1 Fig — Patients who took metformin and insulin glargine U100 were randomized to the empagliflozin or glimepiride groups. Flow-mediated dilation (FMD), blood examination, body weight, and blood pressure were assessed at study baseline and endpoints. (TIF) [file pone.0262831.s001.tif]
