# Supplementary material for: Comparison of the effects of empagliflozin and glimepiride on endothelial function in patients with type 2 diabetes: A randomized controlled study
Source: PLoS One. 2022 Feb 16;17(2):e0262831. doi: 10.1371/journal.pone.0262831 (PMC8849516; doi:10.1371/journal.pone.0262831)
Supplement: S1 Table — (DOCX) [file pone.0262831.s002.docx]

**S1 Table.** **FMD (% ± SD) with treatment for per protocol set.**

|  | **Empagliflozin (*n* = 30)** | **Glimepiride (*n* = 28)** | ***P*-value** |
| --- | --- | --- | --- |
| FMD (0) | 5.49 ± 2.05 | 5.46 ± 2.20 | *P* = 0.96 |
| FMD (12) | 5.30 ± 2.28 | 5.09 ± 1.86 | *P* = 0.71 |
|  | *P* = 0.66 | *P* = 0.49 |  |
| ΔFMD (12) − (0) | −0.19 ± 2.34 | −0.37 ± 2.77 | *P* = 0.79 |

Values are presented as mean ± standard deviation (SD). FMD, flow-mediated dilation; FMD (0) means the baseline FMD value at 0 week. FMD (12) means the FMD value at 12 weeks after additional treatment; Δ indicates the change in the FMD value between 0 and 12 weeks; *P*-values in each row refer to a comparison of FMD values at the baseline and at week 12; *P*-values in each line refer to a comparison of changes in FMD values for both groups. Paired Student’s *t*–tests were used to compare values between two groups, and paired *t*–tests were used to compare values obtained before and after additional treatment.
