# Supplementary material for: Comparison of the effects of empagliflozin and glimepiride on endothelial function in patients with type 2 diabetes: A randomized controlled study
Source: PLoS One. 2022 Feb 16;17(2):e0262831. doi: 10.1371/journal.pone.0262831 (PMC8849516; doi:10.1371/journal.pone.0262831)
Supplement: S2 Table — (DOCX) [file pone.0262831.s003.docx]

**S2 Table.** **Changes in metabolic markers for per protocol set.**

|  | | **Empagliflozin (*n* = 30)** | **Glimepiride (*n* = 28)** |  |
| --- | --- | --- | --- | --- |
| Fasting plasma glucose (mg/dL) [mean ± SD] | | | | |
| Baseline | | 136.9 ± 65.2 | 127.1 ± 52.6 |  |
| Week 12 | | 124.5 ± 52.1 | 119.9 ± 49.1 |  |
|  | | *P* = 0.11 | *P* = 0.47 |  |
| ΔFPG | | −12.4 ± 44.4 | −7.3 ± 52.0 | *P* = 0.69 |
| HbA1c (%) [mean ± SD] | | | | |
| Baseline | | 6.9 ± 1.1 | 6.6 ± 0.7 |  |
| Week 12 | | 6.7 ± 1.1 | 6.4 ± 0.78 |  |
|  | | *P* = 0.001 | *P* = 0.01 |  |
| ΔHbA1c | | −0.22 ± 0.36 | −0.26 ± 0.5 | *P* = 0.75 |
| GA (%) [mean ± SD] | | | | |
| Baseline | | 17.0 ± 3.7 | 16.3 ± 3.4 |  |
| Week 12 | | 16.0 ± 3.2 | 15.7 ± 3.1 |  |
|  | | *P <* 0.001 | *P* = 0.05 |  |
| ΔGA | | −0.97 ± 1.3 | −0.65 ± 1.6 | *P* = 0.42 |
| Renal function | | | | |
| Cr (mg/dL) [mean ± SD] | | | | |
| Baseline | | 0.76 ± 0.16 | 0.73 ± 0.16 |  |
| Week 12 | | 0.79 ± 0.15 | 0.74 ± 0.16 |  |
|  | | *P* = 0.03 | *P* = 0.8 |  |
| eGFR (mL min^−1^ 1.73 m^−2^) [mean ± SD] | | | | |
| Baseline | | 74.9 ± 12.9 | 81.9 ± 19.7 |  |
| Week 12 | | 72.1 ± 11.7 | 81.5 ± 20.2 |  |
|  | | *P* = 0.02 | *P* = 0.8 |  |
| UA (mg/dL) [mean ± SD] | | | | |
| Baseline | | 5.5 ± 1.2 | 5.4 ± 1.5 |  |
| Week 12 | | 4.8 ± 1.2 | 5.7 ± 1.4 |  |
|  | | *P <* 0.001 | *P* = 0.01 |  |
| ΔUA | | −0.64 ± 0.9 | 0.26 ± 0.5 | *P* < 0.001 |
| Body weight (kg) [mean ± SD] | | | | |
| Baseline | | 70.0 ± 11.3 | 69.6 ± 17.1 |  |
| Week 12 | | 69.4 ± 12.0 | 70.8 ± 18.2 |  |
|  | | *P* = 0.22 | *P <* 0.05 |  |
| ΔBody weight | | −0.59 ± 2.5 | 1.2 ± 3.0 | *P* = 0.02 |
| *Waist circumference (cm) [mean ± SD] | | | | |
| Baseline | | 91.6 ± 9.2 | 91.2 ± 15.7 | *P* = 0.27 |
| Week 12 | | 90.9 ± 8.9 | 92.3 ± 15.3 |  |
|  | | *P* = 0.07 | *P* = 0.004 |  |
| ΔWaist circumference | | −0.64 ± 1.8 | 1.1 ± 2.8 | *P* = 0.008 |
| Blood pressure (mmHg) [mean ± SD] | | | | |
| sBP | | | | |
| Baseline | | 129.6 ± 14.9 | 130.1 ± 20.5 |  |
| Week 12 | | 130.2 ± 14.2 | 128.9 ± 19.3 |  |
|  | | *P* = 0.79 | *P* = 0.62 |  |
| ΔsBP | | 0.69 ± 13.7 | −1.3 ± 13.5 | *P* = 0.59 |
| dBP | |  |  |  |
| Baseline | | 80.8 ± 10.0 | 78.0 ± 9.3 |  |
| Week 12 | | 80.6 ± 7.6 | 78.6 ± 12.0 |  |
|  | | *P* = 0.91 | *P* = 0.72 |  |
| ΔdBP | | −0.24 ± 10.8 | 0.64 ± 9.3 | *P* = 0.74 |
| Lipids (LDL-C, HDL-C, TG) (mg/dL) [mean ± SD] | | | | |
| LDL-C | | | | |
| Baseline | | 94.0 ± 26.7 | 89.2 ± 28.1 |  |
| Week 12 | | 107.5 ± 33.0 | 93.9 ± 29.6 |  |
|  | | *P* < 0.001 | *P* = 0.33 |  |
| ΔLDL-C | | 13.5 ± 19.3 | 4.7 ± 25.2 | *P* = 0.14 |
| HDL-C | |  |  |  |
| Baseline | | 54.7 ± 16.0 | 57.4 ± 16.4 |  |
| Week 12 | | 56.0 ± 12.5 | 56.4 ± 13.1 |  |
|  | | *P* = 0.43 | *P* = 0.61 |  |
| ΔHDL-C | | 1.4 ± 9.4 | −1.1 ± 10.9 | *P* = 0.37 |
| TG | |  |  |  |
| Baseline | | 197.7 ± 101.1 | 176.0 ± 131.5 |  |
| Week 12 | | 177.1 ± 86.9 | 185.7 ± 123.7 |  |
|  | | *P* = 0.29 | *P* = 0.8 |  |
| ΔTG | | −20.6 ± 103.8 | 9.6 ± 121.5 | *P* = 0.31 |
| *Body fluid volume (L) [mean ± SD] | | | | |
| Baseline | | 35.8 ± 6.8 | 36.6 ± 8.6 | *P* = 0.69 |
| Week 12 | | 35.4 ± 6.9 | 36.3 ± 8.7 |  |
|  | | *P* = 0.03 | *P* = 0.32 |  |
| ΔBody fluid volume | | −0.33 ± 0.72 | −0.35 ± 1.8 | *P* = 0.94 |
| *Total fat mass (kg) [mean ± SD] | | | | |
| Baseline | | 21.1 ± 6.8 | 20.5 ± 11.0 | *P* = 0.8 |
| Week 12 | | 20.6 ± 6.8 | 21.7 ± 11.6 |  |
|  | | *P* = 0.16 | *P* = 0.02 |  |
| ΔTotal fat mass | | −0.58 ± 2.1 | 1.2 ± 2.3 | *P =* 0.006 |
| Adverse events (%) | | | | |
|  | rash on both arms (3.3%) | | hypoglycemia (3.6%) |  |
| Insulin glargine U100 (U) [mean ± SD] | | | | |
| Baseline | | 9.4 ± 4.6 | 11.7 ± 9.0 | *P* = 0.6* |
| Week 12 | | 8.4 ± 5.1 | 9.6 ± 8.9 |  |
|  | | *P* = 0.02 | *P <* 0.001* |  |
| Δ Insulin glargine U100 | | −1.0 ± 2.4 | −2.1 ± 3.5 | *P* = 0.16* |

*Data of 52 patients (empagliflozin = 26, glimepiride = 26 ) were analyzed.

Values are presented as mean ± standard deviation (SD). Δ indicates the changes in the metabolic markers between 0 and 12 weeks. The *P*-value in each row refers to the comparison of changes in the metabolic markers at baseline and week 12; the *P*-value in each line refers to the comparison of changes in the metabolic markers between the two groups. Paired Student’s *t*–tests were used to compare values between two groups, and paired *t*–tests were used to compare values obtained before and after additional treatment. *Mann-Whitney’s U tests were used. HbA1c, glycated hemoglobin; GA, glycated albumin; Cr, serum creatinine; eGFR, estimated glomerular filtration rate; UA, uric acid; sBP, systolic blood pressure; dBP, diastolic blood pressure; LDL-C, low-density lipoprotein cholesterol; HDL-C, high-density lipoprotein cholesterol; TG, triglycerides
