# Supplementary material for: Comparison of the effects of empagliflozin and glimepiride on endothelial function in patients with type 2 diabetes: A randomized controlled study
Source: PLoS One. 2022 Feb 16;17(2):e0262831. doi: 10.1371/journal.pone.0262831 (PMC8849516; doi:10.1371/journal.pone.0262831)
Supplement: S3 Table — (DOCX) [file pone.0262831.s004.docx]

**S3 Table.** **Changes in metabolic markers for full analysis set.**

|  | Empagliflozin group (*n* = 33) | | | | | Glimepiride group (*n* = 30) | | | | | Difference (Empagliflozin-Glimepiride) | | | | |
| --- | --- | --- | --- | --- | --- | --- | --- | --- | --- | --- | --- | --- | --- | --- | --- |
|  | LS mean | 95%CI | | | P-value vs. baseline | LS mean | 95%CI | | | P-value vs. baseline | LS mean | 95%CI | | | P-value |
| Fasting CPR |  |  |  |  |  |  |  |  |  |  |  |  |  |  |  |
| Baseline | 2.98 | 2.33 | , | 3.62 | - | 2.45 | 1.77 | , | 3.13 | - | 0.53 | -0.41 | , | 1.46 | 0.264 |
| Week 12 | 3.04 | 2.37 | , | 3.71 | 0.851 | 2.79 | 2.10 | , | 3.49 | 0.337 | 0.25 | -0.72 | , | 1.21 | 0.610 |
| ΔF-CPR | 0.06 | -0.62 | , | 0.75 | - | 0.34 | -0.37 | , | 1.06 | - | -0.28 | -1.27 | , | 0.71 | 0.573 |
|  |  |  |  |  |  |  |  |  |  |  |  |  |  |  |  |
| Log U-Alb |  |  |  |  |  |  |  |  |  |  |  |  |  |  |  |
| Baseline | 3.00 | 2.45 | , | 3.55 |  | 2.47 | 1.89 | , | 3.04 |  | 0.54 | -0.26 | , | 1.33 | 0.184 |
| Week 12 | 2.82 | 2.27 | , | 3.38 | 0.237 | 2.42 | 1.84 | , | 3.01 | 0.777 | 0.40 | -0.41 | , | 1.21 | 0.326 |
| ΔU-Alb | -0.18 | -0.48 | , | 0.12 |  | -0.05 | -0.36 |  | 0.27 |  | -0.14 | -0.57 |  | 0.30 | 0.540 |
|  |  |  |  |  |  |  |  |  |  |  |  |  |  |  |  |
| Log L-FABP |  |  |  |  |  |  |  |  |  |  |  |  |  |  |  |
| Baseline | 1.03 | 0.75 | , | 1.31 |  | 0.61 | 0.32 | , | 0.90 |  | 0.42 | 0.02 | , | 0.82 | 0.042 |
| Week 12 | 1.00 | 0.72 | , | 1.28 | 0.757 | 0.67 | 0.37 | , | 0.97 | 0.558 | 0.33 | -0.08 | , | 0.74 | 0.112 |
| ΔLog L-FABP | -0.03 | -0.22 | , | 0.16 |  | 0.06 | -0.14 |  | 0.26 |  | -0.09 | -0.36 |  | 0.19 | 0.524 |
|  |  |  |  |  |  |  |  |  |  |  |  |  |  |  |  |
| HOMA2%B |  |  |  |  |  |  |  |  |  |  |  |  |  |  |  |
| Baseline | 204.05 | 158.19 | , | 249.91 |  | 205.80 | 157.70 | , | 253.90 |  | -1.75 | -68.21 | , | 64.71 | 0.959 |
| Week 12 | 232.21 | 184.50 | , | 279.93 | 0.284 | 274.28 | 224.79 | , | 323.78 | 0.014 | -42.07 | -110.82 | , | 26.68 | 0.228 |
| ΔHOMA2%B | 28.16 | -24.01 | , | 80.33 |  | 68.49 | 14.27 |  | 122.70 |  | -40.33 | -115.57 |  | 34.91 | 0.288 |
|  |  |  |  |  |  |  |  |  |  |  |  |  |  |  |  |
| HOMA2%S |  |  |  |  |  |  |  |  |  |  |  |  |  |  |  |
| Baseline | 21.67 | 14.83 | , | 28.52 |  | 26.38 | 19.20 | , | 33.56 |  | -4.70 | -14.62 | , | 5.22 | 0.349 |
| Week 12 | 21.95 | 14.79 | , | 29.11 | 0.951 | 21.52 | 14.11 | , | 28.94 | 0.295 | 0.42 | -9.89 | , | 10.74 | 0.935 |
| ΔHOMA2%S | 0.27 | -8.56 | , | 9.11 |  | -4.85 | -14.05 |  | 4.34 |  | 5.13 | -7.62 |  | 17.88 | 0.424 |
|  |  |  |  |  |  |  |  |  |  |  |  |  |  |  |  |
| HOMA2IR |  |  |  |  |  |  |  |  |  |  |  |  |  |  |  |
| Baseline | 8.47 | 6.03 | , | 10.91 |  | 6.27 | 3.71 | , | 8.83 |  | 2.20 | -1.33 | , | 5.73 | 0.219 |
| Week 12 | 7.90 | 5.40 | , | 10.39 | 0.546 | 6.61 | 4.01 | , | 9.21 | 0.732 | 1.29 | -2.31 | , | 4.89 | 0.478 |
| ΔHOMA2IR | -0.57 | -2.47 | , | 1.32 |  | 0.34 | -1.63 | , | 2.30 |  | -0.91 | -3.64 | , | 1.82 | 0.506 |

Values are presented as least square means (LS mean) and 95% confidence interval (95%CI). P-values represent the results of linear mixed model analysis with outcome as the dependent variable in FAS. In the model, subjects were the random factors, and time (Baseline or Week 12) and group (Empagliflozin or Glimepiride) and their interaction terms were fixed factors.

F-CPR, fasting C-peptide immunoreactivity; U-Alb, urine albumin; L-FABP, liver-type fatty acid binding protein; HOMA2%B, Homeostasis Model Assessment 2 steady-state beta cell (%B) function; HOMA2%S, Homeostasis Model Assessment 2 insulin sensitivity (%S); HOMA2IR, Homeostasis Model Assessment 2 insulin resistance (IR).
