# Supplementary material for: Comparison of the effects of empagliflozin and glimepiride on endothelial function in patients with type 2 diabetes: A randomized controlled study
Source: PLoS One. 2022 Feb 16;17(2):e0262831. doi: 10.1371/journal.pone.0262831 (PMC8849516; doi:10.1371/journal.pone.0262831)
Supplement: S4 Table — (DOCX) [file pone.0262831.s005.docx]

**S4 Table.** **Changes in metabolic markers for per protocol set.**

|  | **Empagliflozin (*n* =30)** | **Glimepiride (*n* =28)** |  |
| --- | --- | --- | --- |
| **Fasting CPR (ng/mL) [mean ± SD]** | | | |
| Baseline | 3.1 ± 1.7 | 2.5 ± 1.9 |  |
| Week 12 | 3.1 ± 2.2 | 2.8 ± 1.6 |  |
|  | *P* = 0.99 | *P* = 0.34 |  |
| **ΔF-CPR** | −0.003 ± 2.1 | 0.3 ± 1.6 | *P* = 0.55 |
| **Log U-Alb** | | | |
| Baseline | 2.9 ± 1.7 | 2.5 ± 1.6 |  |
| Week 12 | 2.7 ± 1.4 | 2.5 ± 1.5 |  |
|  | *P* = 0.31 | *P* = 0.72 |  |
| **Log L-FABP** | | | |
| Baseline | 0.96 ± 0.85 | 0.6 ± 0.85 |  |
| Week 12 | 0.94 ± 0.7 | 0.69 ± 0.79 |  |
|  | *P* = 0.85 | *P* = 0.66 |  |
| **HOMA2%B** | | | |
| Baseline | 217.8 ± 92.3 | 205.4 ± 105.1 |  |
| Week 12 | 237.8 ± 116.1 | 274.1 ± 194.4 |  |
|  | *P* = 0.32 | *P* = 0.05 |  |
| **ΔHOMA2%B** | 20.0 ±107.2 | 68.7 ± 175.8 | *P* = 0.2 |
| **HOMA2%S** | | | |
| Baseline | 21.5 ± 28.5 | 25.5 ± 16.2 |  |
| Week 12 | 21.9 ± 18.1 | 21.3 ± 14.2 |  |
|  | *P* = 0.54 | *P* = 0.14 |  |
| **ΔHOMA2%S** | 0.4 ± 31.7 | −4.2 ± 14.5 | *P* = 0.09 |
| **HOMA2IR** | | | |
| Baseline | 8.8 ± 8.9 | 6.5 ± 6.1 |  |
| Week 12 | 8.2 ± 8.3 | 6.8 ± 4.4 |  |
|  | *P* = 0.44 | *P* = 0.21 |  |
| **ΔHOMA2%IR** | -0.69 ± 5.5 | 0.27 ± 4.9 | *P* = 0.22 |

Values are represented as means ± standard deviation (SD).

Δ indicates the changes in the FMD values between 0 and 12 weeks.

*P*-values in each row refer to the comparison of FMD values at the baseline and at week 12. The *P*-value in each line refers to the comparison of changes in FMD values for both the groups.

F-CPR, fasting C-peptide immunoreactivity; U-Alb, urine albumin; L-FABP, liver-type fatty acid binding protein; HOMA2%B, Homeostasis Model Assessment 2 steady-state beta cell (%B) function; HOMA2%S, Homeostasis Model Assessment 2 insulin sensitivity (%S); HOMA2IR, Homeostasis Model Assessment 2 insulin resistance (IR).
