# Supplementary material for: Comparison of the effects of empagliflozin and glimepiride on endothelial function in patients with type 2 diabetes: A randomized controlled study
Source: PLoS One. 2022 Feb 16;17(2):e0262831. doi: 10.1371/journal.pone.0262831 (PMC8849516; doi:10.1371/journal.pone.0262831)
Supplement: S5 Table — (DOCX) [file pone.0262831.s006.docx]

**S5 Table. Associations between arterial sclerosis markers and ΔFMD for per protocol set (n = 58).**

|  | **Rawβ** | **Stdβ** | ***P* value** |
| --- | --- | --- | --- |
| Age | 0.010 | 0.050 | 0.7000 |
| Sex | −0.020 | −0.004 | 0.9800 |
| Duration | 0.020 | 0.060 | 0.6700 |
| FMD (0) | −0.740 | −0.610 | ***0.0001 |
| HbA1c (0) | −0.470 | −0.180 | 0.1800 |
| FPG (0) | −0.005 | −0.110 | 0.4000 |
| GA (0) | −0.170 | −0.240 | 0.0700 |
| BW (0) | 0.030 | 0.180 | 0.1800 |
| BMI (0) | 0.120 | 0.200 | 0.1500 |
| UA (0) | 0.310 | 0.160 | 0.2200 |
| LDL (0) | −0.020 | −0.230 | 0.0900 |
| HDL (0) | −0.040 | −0.260 | 0.0500 |
| TG (0) | 0.004 | 0.200 | 0.1100 |
| Past smoker | −0.790 | −0.150 | 0.2500 |
| sBP (0) | −0.030 | −0.180 | 0.1700 |
| dBP (0) | −0.060 | −0.210 | 0.1100 |
| ΔHbA1c | −1.850 | −0.310 | *0.0200 |
| ΔGA | −0.160 | −0.100 | 0.4800 |
| ΔFPG | −0.005 | −0.090 | 0.4900 |
| ΔBW | −0.330 | −0.380 | **0.0030 |
| ΔLDL-C | 0.010 | 0.110 | 0.4300 |
| ΔHDL-C | 0.080 | 0.310 | *0.0200 |
| ΔTG | −0.009 | −0.380 | **0.0030 |
| ΔsBP | 0.010 | 0.060 | 0.6300 |
| ΔdBP | 0.050 | 0.180 | 0.18 |
| ARB/ACE-I | 0.410 | 0.070 | 0.59 |
| Statin | 0.340 | 0.070 | 0.62 |

Values are presented as means. **P* < 0.05, ** *P* < 0.01, and ****P* < 0.001 indicate a significant association between arterial sclerosis markers and ΔFMD.

(0), baseline; FMD, flow-mediated dilation; HbA1c, glycated hemoglobin; FPG, fasting plasma glucose; GA, glycated albumin; BW, body weight; BMI, body mass index; UA, uric acid; LDL, low-density lipoprotein; HDL, high-density lipoprotein; TG, triglycerides; sBP, systolic blood pressure; dBP, diastolic blood pressure; LDL-C, LDL cholesterol; HDL-C, HDL cholesterol; ARB, angiotensin receptor blocker; ACE-I, angiotensin converting enzyme inhibitor
