# Supplementary material for: Comparison of the effects of empagliflozin and glimepiride on endothelial function in patients with type 2 diabetes: A randomized controlled study
Source: PLoS One. 2022 Feb 16;17(2):e0262831. doi: 10.1371/journal.pone.0262831 (PMC8849516; doi:10.1371/journal.pone.0262831)
Supplement: S6 Table — (DOCX) [file pone.0262831.s007.docx]

**S6 Table. Association between anti-diabetic therapy and ΔFMD in the empagliflozin and glimepiride groups for per protocol set.**

Empagliflozin (n = 30)

| **Therapy** | **Rawβ** | **Stdβ** | ***P*-value** |
| --- | --- | --- | --- |
| Metformin | −0.18 | −0.070 | 0.78 |
| DPP-4 inhibitor | −0.84 | −0.330 | 0.19 |
| SGLT2-inhibitor | 0.02 | 0.004 | 0.98 |
| Sulfonylurea | 1.00 | 0.300 | 0.27 |
| Insulin | 0.82 | 0.180 | 0.39 |

Exception: glinide (n = 1)

Glimepiride (n = 28)

|  | **Rawβ** | **Stdβ** | ***P* value** |
| --- | --- | --- | --- |
| Metformin | −0.18 | −0.05 | 0.84 |
| DPP-4 inhibitor | 0.91 | 0.30 | 0.22 |
| Sulfonylurea | 0.95 | 0.24 | 0.47 |
| α-glucosidase inhibitor | −1.07 | −0.20 | 0.57 |
| Insulin | −0.41 | −0.08 | 0.75 |

Exception: glinide, SGLT2-inhibitor, and GLP-1 analogue (n = 1)

FMD, flow-mediated dilation
