# Supplementary material for: Comparison of the effects of empagliflozin and glimepiride on endothelial function in patients with type 2 diabetes: A randomized controlled study
Source: PLoS One. 2022 Feb 16;17(2):e0262831. doi: 10.1371/journal.pone.0262831 (PMC8849516; doi:10.1371/journal.pone.0262831)
Supplement: S1 Protocol — (DOC) [file pone.0262831.s008.doc]

**研究実施計画書**

研究課題名称

「2型糖尿病におけるエンパグリフロジンの血管内皮機能へ

の効果についてのランダム化比較試験」

研究代表者：茅ヶ崎市立病院　代謝内分泌内科　佐藤　忍

臨床研究実施予定期間：承認後～2019年3月31日（3年間）

作　成　日 ：2016年3月24日

**1．研究課題名**

「2型糖尿病におけるエンパグリフロジンの血管内皮機能への効果についてのランダム化比較試験」

**2．実施責任者**

　茅ヶ崎市立病院　代謝内分泌内科　佐藤　忍

**3．研究分担者**

茅ヶ崎市立病院　代謝内分泌内科1)

横浜市立大学大学院医学研究科 分子内分泌糖尿病学2)

田村 遥1,2)　近藤 義宣1,2)　井上 雄一郎1) 鈴木 陽一1)　吉田 富美菜1)

井上 幸奈1)　宮﨑 裕子1) 寺内 康夫2)

**4．研究背景と意義**

糖尿病患者では、非糖尿病患者に比べて冠動脈疾患の頻度が上昇し、冠動脈疾患の既往がない糖尿病患者の心筋梗塞発症率は、非糖尿病患者で心筋梗塞の既往がある者の再発率とほぼ同等であるとされている。^1)^また、内臓脂肪蓄積により、アディポサイトカインの分泌異常、脂肪組織の慢性炎症が起こり、インスリン抵抗性を惹起し、インスリン抵抗性は血管内皮機能障害と関連する。^2)3)^血管内皮機能障害は長期的な動脈硬化性病変、心血管イベントの発生率を予測することができ、^4)^Flow Mediated Dilation （FMD）によって非侵襲的に評価することができる。^5)^

2015年、国内でSGLT-2阻害薬が発売され、エンパグリフロジンは心血管ハイリスクの2型糖尿病患者においてプラセボ群と比較して、心血管による死亡、心血管イベント、全死亡の発症率を低下させたと報告された。^6)^また、ダパグリフロジンは内臓脂肪量、皮下脂肪量共に減少させるが、特に内臓脂肪を顕著に減少させると報告された。^7)^

SGLT-2阻害薬は内臓脂肪減少による血管内皮機能障害改善の効果、早期動脈硬化予防治療介入により、心血管死亡を減少させる効果が期待され、SGLT-2阻害薬の血管内皮機能への効果をFMDで評価する。

**5．研究目的**

　2型糖尿病患者におけるエンパグリフロジンの血管内皮機能への効果を検討する。

**6．研究方法**

（1）研究対象者

以下の条件を満たす2型糖尿病患者。

・選択基準

1. 年齢　20歳以上　80歳未満
2. BMI　45kg/m2以下

・除外基準

1. 重篤な腎機能障害（eGFR<45ml/min/1.73m2）を有する患者
2. 重篤な肝障害を有する患者
3. 24週以内発症の心血管、脳血管障害を有する患者
4. ステロイド使用中の患者
5. 担癌患者
6. 重症感染症、重篤な外傷を有する患者
7. 妊娠、妊娠の可能性がある患者
8. 使用薬剤にアレルギーを有する、もしくは服用禁忌な患者
9. その他試験担当医師が本試験に不適切と判断した患者

（2）対象者への同意取得方法

本研究開始前に研究担当医師は、｢ヘルシンキ宣言｣に基づいたインフォームド・コンセントを被験者に対して個別に行う。本研究の目的、方法、危険性、結果の報告方法などについて理解を得たうえで、本研究への参加の同意を文書にて得る。対象者が本研究に同意しない場合でも不利益を受けないこと、同意した場合でも随時これを撤回できること、対象者の人権保護など必要な事項について対象者に十分説明する。

なお、対象者の同意に影響を及ぼすような実施計画の変更が行われるときは、速やかに対象者に情報提供し、調査に参加するか否かについて意思を予め確認するとともに、事前に同意説明文書等の改訂を行い、対象者の再同意を得る。

（3）研究デザイン

茅ヶ崎市立病院　代謝内分泌内科に教育入院し、2型糖尿病と診断された患者に対し、非盲検無作為化比較試験を行う。教育入院でメトホルミン+基礎インスリン療法で退院、代謝改善前後でのFMD測定をする。代謝状態の安定した12週間後に、グリメピリド0.5mg投与あるいはエンパグリフロジン10mgを投与開始する。追加治療開始12週後FMD測定をする。

1. 研究アウトライン

**入院中**

同意書取得

FMD測定

副次評価項目測定

メトホルミン+グラルギン眠前投与で退院

↓

**11±2週間後**

FMD測定

副次評価項目測定

割り付け

↓

**12±2週間後**

グリメピリド0.5mgあるいはエンパグリフロジン10mg投与開始

ランダム化後、12週は治療を変更しない。

ただし空腹時血糖が200mg/dl以上が続く時は1週間毎に基礎インスリン1単位ずつ増量可、空腹時血糖が90mg/dl以下が続く時は1週間毎に基礎インスリン1単位ずつ減量可とする。

↓

**24±2週間後**

FMD測定

副次評価項目測定

（5）評価項目調査・測定項目及び方法

1)　主要評価項目

1追加治療開始前と追加治療開始12週後のFMD％の変化

2) 副次評価項目

1. 入院時と追加治療開始時のFMD%の変化
2. 空腹時血糖の変化
3. 食後2時間血糖の変化
4. HbA1cの変化
5. GAの変化
6. 空腹時CPRの変化（CPR-index、HOMA-β2、HOMA-R2算出）
7. 自己血糖測定結果
8. 腎機能の変化（クレアチニン、eGFR、シスタチンC、Cys-eGFR）
9. 尿中アルブミンの変化
10. 尿中L－FABPの変化
11. 尿酸代謝の変化
12. 体重の変化
13. ウエストの変化
14. ウエストヒップ比の変化
15. 体組成の変化
16. 血圧、脈拍の変化
17. 脂質プロファイル（TC、TG、HDL-C、LDL-C）の変化
18. インスリン使用量の変化
19. 心機能（心臓超音波検査）
20. 有害事象

|  | 入院時 | 追加投与前 | 追加投与12  週 |
| --- | --- | --- | --- |
| 血管内皮機能検査(FMD%) | ● | ● | ● |
| 空腹時血糖(mg/dl) | ● | ● | ● |
| 食後2時間血糖(mg/dl) | ● | ● | ● |
| HbA1c(%, NGSP) | ● | ● | ● |
| グリコアルブミン(%) | ● | ● | ● |
| インスリン分泌能指標(空腹時Cペプチド) | ● | ● | ● |
| 腎機能検査 | ● | ● | ● |
| 尿中アルブミン(mg/gCr) | ● | ● | ● |
| 尿中L型脂肪酸結合蛋白(L-FABP)(μg/gCr) | ● | ● | ● |
| 尿酸(mg/dl) | ● | ● | ● |
| 身長(cm) | ● |  |  |
| 体重(kg) | ● | ● | ● |
| ウエスト径(cm) | ● | ● | ● |
| ウエストヒップ比 | ● | ● | ● |
| 体組成(InBody®) | ● |  | ● |
| 血圧(mmHg) | ● | ● | ● |
| 脈拍(回/分) | ● | ● | ● |
| インスリン使用量(単位) | ● | ● | ● |
| 心臓超音波 | ● |  | ● |
| 脂質プロファイル (TC(mg/dl), TG(mg/dl), HDL-C(mg/dl), LDL-C(mg/dl)) | ● | ● | ● |
| 有害事象 | ● | ● | ● |

（6）有害事象及び副作用

メトホルミン+基礎インスリン療法にグリメピリド、エンパグリフロジンを追加投与することで、血糖コントロールが改善し、低血糖が出現する可能性がある。低血糖が出現した場合にはブドウ糖内服などの適切な対処法を患者に指導することでリスク回避を行い、担当医師の判断で適切な治療を行う。

また、エンパグリフロジンに特徴的な副作用として脱水（0.1％）、膀胱炎、尿路感染、外陰部膣カンジダ症（0.1-5％）報告があり、投与前には十分な水分補給、清潔を指導する。本研究の期間中あるいは終了後に健康障害が生じた場合には、医師が適切な治療を行う。

**7．記録等の保存および個人情報保護の方法**

個人が特定されることがないように、分析を行なう前に、氏名、性別、年齢を削除し、登録番号をつけ、匿名化を行う。匿名化は、茅ヶ崎市立病院内にて行い、対象者個人と番号の対応表は、個人情報管理者（栗山仁）により厳重に管理される。研究責任者、分担者が茅ヶ崎市立病院外に調査票を持ち出す場合は、匿名化された調査票のみとする。さらに、研究分担者が本研究の結果を使う場合も、個人情報の取り扱い、処理方法などは対象者の意思を尊重し、厳重に管理される。また、個人情報は、本研究の目的以外には使用せず、研究代表者の研究室内にて施錠できる保管庫を用いて5年間厳重に保管し、第三者に漏れないよう厳重に管理される。その後シュレッダーにより処理できるものは処理し、その他のものは適切な処理をしてデータの復旧ができない状態にする。

研究結果は、本研究の趣旨に沿う学術論文や学会等で発表されることが前提となる。この場合、名前や個人を認識できる情報は一切公表せず、プライバシーは保護される。

また、研究責任者は、調査等の実施に係る必須文書を保存し、研究発表後5年後に破棄する。なお、調査の結果は、分析の後、被験者本人に開示・返却することを前提とし、その結果は診療を行なう際の参考資料として用いる。

**8．倫理的配慮**

本研究は、ヘルシンキ宣言に基づく倫理的原則、臨床研究に関する倫理指針および茅ヶ崎市立病院の倫理規約に従い本研究計画書を遵守して実施する。

（１）倫理委員会による審査と承認

　　　本研究の実施に先立ち、茅ヶ崎市立病院倫理委員会において審査を行い、倫理委員会が研究の実施を承認した後に本研究を実施する。なお、研究期間を通じ倫理委員会の審査の対象となる文章が追加、更新、または改訂された場合（軽微な追加、更新または改訂は除く）にも同様の審査を受けるものとする。

（２）プライバシーの保護

症例報告書の作成、対象者のデータの取り扱い等については対象者のプライバシーの保護に配慮し、個人が特定できないように対象者のデータは全て暗号化する。すなわち、対象者の氏名やイニシャルは使用せず、対象者識別コードで特定するものとする。

**9．対象者の費用負担**

　通常の診察範囲内で行われる血液生化学検査及び生理検査は、対象者の負担とする。その他の調査項目についても対象者の費用負担は一切発生しない。また、通院にかかる交通費は、対象者の本人負担とする。

**10．臨床研究実施期間**

　承認後から2019年3月31日

**11．研究結果の公表**

本研究で得られた研究成果の発表に関しては、糖尿病学会等で発表し、その後原著論文として雑誌に投稿する。

**12．研究に関連した参考資料・文献リスト**

1. Mortality from coronary heart disease in subjects with type 2 diabetes and in nondiabetic subjects with and without prior myocardial infarction

N Engl J Med 1998;339:229-34.

2. Adiponectin and adiponectin receptors in insulin resistance,diabetes,and the metabolic syndrome

J.Clin.Invest.116:1784-1792(2006)

3.Endothelial Dysfunction Is Detectable in Young Normotensive First-Degree Relatives of Subjects With Type 2 Diabetes in Association With Insulin Resistance

Circulation.2000;101:1780-1784.

4.Prognostic Impact of Coronary Vasodilator Dysfunction on Adverse Long-Term Outcome of Coronary Heart Disease

Circulation.101:1899-1906,2000

5.Type 2 diabetes is associated with impaired endothelium-dependent,flow-mediated dilation,but impaired glucose metabolism is not;The Hoorn Study.

Atherosclerosis.174(1):49-56,2004

6.Empagliflozin,Cardiovascular Outcomes,and Mortality in type2 Diabetes.

N Engl J Med.373(22):2117-28,2015

7.Dapagliflozin maintains glycaemic control while reducing weight and body fat mass over 2 years in patients with type2 diabetes mellitus inadequately controlled on met

Diabetes, Obesity and Metabolism 16:159-169,2014
