# Supplementary material for: Comparison of the effects of empagliflozin and glimepiride on endothelial function in patients with type 2 diabetes: A randomized controlled study
Source: PLoS One. 2022 Feb 16;17(2):e0262831. doi: 10.1371/journal.pone.0262831 (PMC8849516; doi:10.1371/journal.pone.0262831)
Supplement: S2 Protocol — (DOC) [file pone.0262831.s009.doc]

**Original protocol translated**

Research proposal title

A randomized comparison of the effect of empagliflozin on endothelial function in type 2 diabetes

Principal Investigator: Shinobu Satoh, Department of Metabolic Endocrinology, Chigasaki Municipal Hospital

Anticipated Clinical Research Period: Post-Approval to March 31, 2019 (Three Years)

Creation date: March 24, 2016

**1. Study title**

A randomized comparison of the effect of empagliflozin on endothelial function in type 2 diabetes

**2. Principal investigator**

　Shinobu Satoh, Department of Metabolic Endocrinology, Chigasaki Municipal Hospital

**3. Co-investigators**

Department of Metabolic Endocrinology, Chigasaki Municipal Hospital 1).

Yokohama City University Graduate School of Medicine, Molecular Endocrinology and Diabetology 2).

Haruka Tamura 1,2) Yoshinobu Kondo 1,2) Yuichiro Inoue 1) Yoichi Suzuki 1) Fumina Yoshida 1)Yukina Inoue 1) Yuko Miyazaki 1) Yasuo Terauchi 2)

**4. Research background and significance**

The incidence rate of coronary artery diseases has been shown to be higher in diabetic patients than in non-diabetic patients. Further, the incidence of myocardial infarction in diabetic patients without a history of coronary artery disease has been shown to be similar to that of non-diabetic patients with a history of myocardial infarction^1)^. Visceral adiposity has been shown to result in abnormal secretions of adipocytokines and chronic inflammation of adipose tissue, which induces insulin resistance. Insulin resistance has been correlated with vascular endothelial dysfunction^2)3)^, which in turn, has been shown to predict the incidence of long-term arteriosclerotic lesions and cardiovascular events^4)^. Importantly, endothelial dysfunction can be non-invasively assessed by flow mediated dilation (FMD)^5)^.

Sodium-glucose cotransporter-2 **(**SGLT-2) inhibitors were launched in Japan in 2015, and empagliflozin was reported to reduce cardiovascular death, cardiovascular events, and overall mortality rates in type 2 diabetes patients with high cardiovascular risk than in placebo-treated patients^6)^. Dapagliflozin has also been reported to reduce subcutaneous fat mass, and particularly, visceral fat mass^7)^.

SGLT-2 inhibitors are expected to improve vascular endothelial dysfunction by reducing visceral fat and cardiovascular mortality with early arteriosclerosis prevention treatment interventions. Here, we propose to evaluate the effects of SGLT-2 inhibitors on vascular endothelial function using FMD.

**5. Research objective**

　To investigate the effect of empagliflozin on vascular endothelial function in patients with type 2 diabetes.

**6. Research methods**

(1) Study subjects

Patients with type 2 diabetes who meet the following conditions:

・ Selection criteria

1. Age between 20 and 80 years
2. BMI equal to or less than 45 kg / m^2^

・ Exclusion criteria

1. Patients with serious renal dysfunction (estimated glomerular filtration rate [eGFR] < 45 mL/min/1.73 m^2^).
2. Patients with serious hepatic disorders
3. Patients with cardiovascular or cerebrovascular disorders occurring within 24 weeks of the trial
4. Patients using steroids
5. Patients with tumors
6. Patients with severe infections or injuries
7. Patients who are or may be pregnant
8. Patients who are allergic to or have contraindications for the use of drugs
9. Other patients considered inadequate for the study by the investigator

(2) Methods for obtaining informed consent from participants

Based on the Declaration of Helsinki, the investigator will obtain informed consent from each patient immediately before the start of the trial. Informed consent for participation will be obtained in writing once the patients have understood the purpose, methods, hazards, and result reporting methods of this study. Full explanations shall be provided to the patients regarding the following matters: the patient will not suffer any disadvantages even if they did not consent to the study; the patient may withdraw consent at any point during the trial even if they had originally consented; and the patient’s human rights protection and other necessary matters.

The patient shall also be promptly informed of any changes in the implementation plan that may affect their consent. In such cases, the patient’s intention to participate in the investigation will be confirmed and the consent form will be revised in advance so that consent may be obtained from the patient a second time.

(3) Research Design

An open-label, randomized controlled trial will be conducted on patients diagnosed with type 2 diabetes mellitus and admitted to the Department of Metabolic Endocrinology at Chigasaki Municipal Hospital. FMD measurements will be carried out at the time of admission as well as at the time of discharge following metformin and basal insulin therapy, and before and after metabolic improvement. A total of either 0.5 mg of glimepiride or 10 mg of empagliflozin will be administered after 12 weeks of stable metabolic status. FMD measurements will be taken 12 weeks after the start of additional treatment.

1. Research outline

**During hospitalization**

Consent form obtained

FMD measurement

Secondary endpoint measurement

Discharge with metformin + glargine administration prior to sleep

↓

**After 11 ± 2 weeks**

FMD measurement

Secondary endpoint measurement

Allocation

↓

**After 12 ± 2 weeks**

Start administration of 0.5 mg glimepiride or 10 mg empagliflozin

After randomization, treatment will not be changed for 12 weeks.

However, dosage may be increased by 1 IU of basal insulin every week if the fasting blood glucose continues to be 200 mg / dL or more, and decreased by 1 IU of basal insulin every week if the fasting blood glucose continues to be 90 mg / dL or less.

↓

**After 24 ± 2 weeks**

FMD measurement

Secondary endpoint measurement

(5) Endpoint investigation / parameters and methods

1) Primary endpoint

1 Change in FMD% before the start of additional treatment and 12 weeks after the start of additional treatment

2) Secondary endpoints

1. Change in FMD% between admission and start of additional treatment
2. Changes in fasting blood glucose
3. Changes in 2-hour postprandial blood glucose
4. Changes in HbA1c
5. Changes in glycatedalbumin (GA)
6. Changes in fasting C-peptide immunoreactivity [CPRs] (CPR-index, HOMA-β2, HOMA-R2 calculated)
7. Blood glucose self-monitoring results
8. Changes in renal function (creatinine, eGFR, cystatin C, Cys-eGFR)
9. Changes in urinary albumin
10. Changes in urinary Liver-type fatty acid binding protein (L-FABP)
11. Changes in uric acid metabolism
12. Changes in body weight
13. Changes in waist diameter
14. Changes in waist-hip ratio
15. Changes in body composition
16. Changes in blood pressure and pulse rate
17. Changes in lipid profiles (total cholesterol [TC], triglycerides [TG], high-density lipoprotein cholesterol [HDL-C], low-density lipoprotein cholesterol [LDL-C])
18. Changes in insulin use
19. Cardiac function (cardiac ultrasound inspection)
20. Adverse events

|  | During hospitalization | Before additional dose | 12 weeks after additional dose |
| --- | --- | --- | --- |
| Vascular endothelial function test (FMD%) | ● | ● | ● |
| Fasting blood glucose (mg/dL) | ● | ● | ● |
| Two-hour postprandial blood glucose (mg/dL) | ● | ● | ● |
| HbA1c (%, NGSP) | ● | ● | ● |
| Glycatedalbumin (%) | ● | ● | ● |
| Insulin secretory capacity index (fasting C-peptide) | ● | ● | ● |
| Renal function test | ● | ● | ● |
| Urinary albumin (mg/gCr) | ● | ● | ● |
| Urinary Liver-type fatty acid binding protein (L-FABP) (μg/gCr) | ● | ● | ● |
| Uric acid (mg/dL) | ● | ● | ● |
| Height (cm) | ● |  |  |
| Body weight (kg) | ● | ● | ● |
| Waist circumference (cm) | ● | ● | ● |
| Waist/hip ratio | ● | ● | ● |
| Body composition (InBody®) | ● |  | ● |
| Blood pressure (mmHg) | ● | ● | ● |
| Pulse (beats/min) | ● | ● | ● |
| Insulin Use (Units) | ● | ● | ● |
| Echocardiogram | ● |  | ● |
| Lipid profiles (TC (mg/dL), TG (mg/dL), HDL-C (mg/dL), LDL-C (mg/dL)) | ● | ● | ● |
| Adverse events | ● | ● | ● |

(6) Adverse events and side effects

The additional administration of glimepiride and empagliflozin to metformin + basal insulin therapy may improve glycemic control and lead to the appearance of hypoglycemia. Risk avoidance shall be performed by instructing the patient to take appropriate measures, such as internal glucose administration if hypoglycemia appears. Further, appropriate treatment shall be provided at the discretion of the physician in charge.

Previously reported characteristic side effects of empagliflozin include dehydration (0.1%), cystitis, urinary tract infection, and vulvovaginal candidiasis (0.1-5%). Thus, adequate hydration and cleanliness shall be advised before administration. The physician will provide appropriate treatment if health problems occur during or after the study period.

**7. Preservation of records and personal information protection methods**

To prevent individuals from being identified, anonymization shall be conducted prior to analysis. To this end, the patient’s name, sex, and age shall be deleted and a registration number assigned to each individual. Anonymization shall carried out at Chigasaki City Hospital, and the document linking the patient’s identity to their assignation number shall be strictly controlled by the personal information manager (Hitoshi Kuriyama). Only an anonymized questionnaire shall be used when a research manager or co-investigator brings a questionnaire out of Chigasaki Municipal Hospital. The handling of personal information and processing methods will also be strictly controlled. The patients’ wishes shall be respected when the co-investigators use the results of this study. Personal information shall be strictly kept in locked storage in the principal investigator’s laboratory for 5 years using archives that are not used for any purposes other than for this study and strictly managed to avoid leakage to third parties. Later, documents shall be destroyed by shredding and other forms of data shall also be appropriately treated so that the data cannot be restored.

It is assumed that the research results will be presented in academic papers and conferences in accordance with the purpose of this study. In such cases, no names or individually-recognizable information will be published, and privacy will be protected.

The research manager shall also store essential documents related to the conducting of surveys, etc., and destroy them 5 years after the publication of the study. It is presumed that the investigation results will be disclosed and returned to the participants after analysis, with the results used as a reference for conducting medical care.

**8. Ethical considerations**

This study will be conducted in accordance with this research proposal plan and the ethical principles based on the Declaration of Helsinki, ethical guidelines for clinical research and the Ethical Code of Chigasaki Municipal Hospital.

(1) Review and approval by the ethics committee

　　　A review shall be conducted at the Chigasaki Municipal Hospital Ethics Committee prior to the implementation of this study, which shall be conducted following approval from the ethics committee. A similar review shall be conducted if additions, updates, or revisions are made (except for minor additions, updates, or revisions) to texts that were subject to review by the ethics committee during the research period.

(2) Protection of privacy

Patient privacy protection shall be taken into consideration during the preparation of the case report form and handling of patients’ data. All patient data shall be coded so that the individual cannot be identified. Therefore, patients’ name and initials shall not be used, and they shall only be able to be identified by the patient-specific identification code.

**9. Patient expenses and burdens**

　Burdens on the patients include blood chemistry tests and physiological examinations performed as part of the standard physical examinations. No expenses shall be borne by the patients for any other research items. Travel expenses for outpatient treatment shall be borne by the patients themselves.

**10. Duration of clinical research**

　From the data of approval until March 31, 2019

**11. Publication of study results**

The results obtained from this study will be presented at diabetes conferences and published in academic journals as original articles.

**12. References**

1. Mortality from coronary heart disease in subjects with type 2 diabetes and in nondiabetic subjects with and without prior myocardial infarction

N Engl J Med. 1998;339:229-234.

2. Adiponectin and adiponectin receptors in insulin resistance, diabetes, and the metabolic syndrome

J Clin Invest. 2006;116:1784-1792.

3. Endothelial dysfunction is detectable in young normotensive first-degree relatives of subjects with type 2 diabetes in association with insulin resistance

Circulation. 2000;101:1780-1784.

4. Prognostic impact of coronary vasodilator dysfunction on adverse long-term outcome of coronary heart disease

Circulation. 2000;101:1899-1906.

5. Type 2 diabetes is associated with impaired endothelium-dependent, flow-mediated dilation, but impaired glucose metabolism is not; The Hoorn Study.

Atherosclerosis. 2004;174(1):49-56.

6. Empagliflozin, cardiovascular outcomes, and mortality in type 2 diabetes.

N Engl J Med. 2015;373(22):2117-2128.

7. Dapagliflozin maintains glycemic control while reducing weight and body fat mass over 2 years in patients with type 2 diabetes mellitus inadequately controlled on metformin

Diabetes, Obesity and Metabolism. 2014;16:159-169.
